# Comparison of a human neuronal model proteome upon Japanese encephalitis or West Nile Virus infection and potential role of mosquito saliva in neuropathogenesis

**DOI:** 10.1371/journal.pone.0232585

**Published:** 2020-05-06

**Authors:** Benoit Besson, Justine Basset, Sandrine Gatellier, Hélène Chabrolles, Thibault Chaze, Véronique Hourdel, Mariette Matondo, Nathalie Pardigon, Valérie Choumet

**Affiliations:** 1 Institut Pasteur, Environment and Infectious Risks Unit, Arbovirus Group, Paris, France; 2 Institut Pasteur, Plateforme Protéomique, Unité de Spectrométrie de Masse pour la Biologie (MSBio), Centre de Ressources et Recherches Technologiques (C2RT), USR CNRS, Paris, France; University of Hong Kong, HONG KONG

## Abstract

Neurotropic flavivirus Japanese encephalitis virus (JEV) and West Nile virus (WNV) are amongst the leading causes of encephalitis. Using label-free quantitative proteomics, we identified proteins differentially expressed upon JEV (gp-3, RP9) or WNV (IS98) infection of human neuroblastoma cells. Data are available via ProteomeXchange with identifier PXD016805. Both viruses were associated with the up-regulation of immune response (IFIT1/3/5, ISG15, OAS, STAT1, IRF9) and the down-regulation of SSBP2 and PAM, involved in gene expression and in neuropeptide amidation respectively. Proteins associated to membranes, involved in extracellular matrix organization and collagen metabolism represented major clusters down-regulated by JEV and WNV. Moreover, transcription regulation and mRNA processing clusters were also heavily regulated by both viruses. The proteome of neuroblastoma cells infected by JEV or WNV was significantly modulated in the presence of mosquito saliva, but distinct patterns were associated to each virus. Mosquito saliva favored modulation of proteins associated with gene regulation in JEV infected neuroblastoma cells while modulation of proteins associated with protein maturation, signal transduction and ion transporters was found in WNV infected neuroblastoma cells.

## Introduction

Arboviral diseases continue to represent a major burden for society, with both health and economic consequences. Japanese encephalitis virus (JEV) and West Nile virus (WNV), two closely-related *Flavivirus* [1], are the most important cause of encephalitis amongst arboviruses, leading to large outbreaks in Asia for the former, and is the principal cause of epidemic encephalitis in the United States, for the latter [2]. Other mosquito-borne flaviviruses can also display neurotropic features such as dengue virus in rare cases, Saint-Louis encephalitis virus (SLEV) or the recently emerged Zika virus as well as the tick-borne encephalitis virus (TBE) [3]. As recently highlighted in several studies, both JEV and WNV are presenting high risks of spillover in immunologically naive human populations: native mosquitoes from both Europe and Northern America have been shown to be competent vectors for JEV [4] [5] and WNV is currently spreading in the Eastern and Southern parts of Europe [6]. JEV and WNV are transmitted to vertebrate hosts such as birds (and domestic swine for JEV) by *Culex* mosquitoes in an enzootic cycle and humans (and horses) are considered dead-end hosts as they may develop symptomatic infection while they cannot transmit the disease [7].

During infection, viruses hijack various host cell pathways to promote their own replication and survival while cells activate various antiviral responses [8]. Hence, the resulting host-virus interactions determines the outcome of viral infection and disease progression [9]. Recent technological advances in proteomics have led to a better understanding of the complex nature of virus-host interactions through large-scale screening approaches, using so far mostly two-dimensional differential gel electrophoresis (2D-DIGE) and mass spectrometry (MS) [11–13]. Two proteomic studies performed in HeLa cells [10] or mouse brains and neuroblastoma cells [11] reported several confirmed hits and pathways as being modulated during JEV infection. However, none were consistently identified in both studies. One laboratory reported several confirmed hits and pathways modulated in WNV-infected Vero cells [12] and mouse brains [13] but none of them corroborated those found for JEV. In the first study using label-free MS, phosphorylation of the spliceosome, ErbB, MAPK, NF-κB and mTOR signaling pathways were found to be highly regulated during WNV infection of human glial U251 cells [14,15]. Yet, there is no proteomic study comparing the host response to JEV and WNV infection in an identical experimental model. The only large-scale screening providing a wide comparison between JEV and WNV infection was performed using microarrays and based on infected mouse brain [16,17]. Viral infection induced a modulation of the expression of genes associated with interferon signaling, the immune system, inflammation, cell death, survival, glutamate signaling and tRNA charging, the latter being specific to flaviviruses.

While most of the infections by neurotropic flaviviruses are asymptomatic or showing mild symptoms such as fever, headache or gastrointestinal symptoms, the most devastating outcome–encephalitis–occurs only in <1% of cases [14]. The question of JEV or WNV capacity to successfully infect the brain and induce acute encephalitis in humans remains to be clarified. To gain access to the central nervous system (CNS), most viruses have to cross the blood-brain barrier (BBB). In the case of neurotropic flaviviruses, the mechanisms involved are not completely understood yet [18], but disruption of the BBB appears to be a likely possibility [19]. Interestingly, studies have shown that mosquito saliva was discharged in the blood vessel [20] and therefore could potentially reach the BBB. Mosquito saliva has already been well-established as a potent enhancer of various arbovirus infections [21]. In the case of dengue virus, mosquito bite enhances viral pathogenesis [22] and mosquito saliva was suggested to favor keratinocyte infection and modify the host immune response [23,24]. Regarding neurotropic flaviviruses, the role of saliva was confirmed for WNV [23,24], not shown for SLEV [25,26] and remains to be established for JEV. WNV inoculation in mice after mosquito feeding led to a higher viremia and an accelerated neuroinvasion [24]. However, the role of saliva has been mostly studied on the site of infection or/and with a low quantity of saliva, mimicking a single mosquito bite event [27]. Spot feeding by uninfected mosquitoes prior to viral inoculation of mice has been shown to enhance WNV viremia in a dose-dependent manner [5]. Moreover, mice inoculated by infected mosquito bite had faster viral spread to peripheral and central nervous system tissues than mice inoculated by needle [5]. Yet in endemic areas individuals are regularly subjected to several mosquito bite events. Taken together, it is thus possible that in highly bitten individuals, saliva proteins may reach the BBB and CNS from the blood stream, independently or bound to the virus [5] and affect viral replication there.

In order to better understand the pathophysiological processes involved during neurotropic flavivirus infection, we compared the profiles of protein expression in JEV or WNV-infected human neuroblastoma cells using label-free quantification mass-spectrometry (MS). We identified numerous cellular factors up- or down-regulated during JEV or WNV infection. Interestingly, a consequent number of proteins was similarly regulated by both viruses. Finally, we investigated the potential role of mosquito saliva on neuroblastoma cell infection by both JEV and WNV. Surprisingly, we found a strong effect of mosquito saliva on the human neuroblastoma proteome for either infection, although neither JEV nor WNV replication was affected in the cells infected.

## Methods

### Mosquito rearing and salivary gland extraction

Colonies of *Cx*. *pipiens*, a competent vector for both JEV and WNV [5], were established from field isolates and reared as previously described [5]. Briefly, the eggs were hatched in tap water and the *larvae* were fed with brewer’s yeast tablets and cat food. Adults were maintained at 27°C, 80% relative humidity with continuous access to 10% sucrose solution and a light/dark cycle of 12 h/12 h.

Five days after hatching, female mosquitoes were anesthetized at 4°C. 100 salivary glands (SG) were dissected and placed in 100 μL of PBS 1X. Salivary glands extracts (SGE) were prepared by sonicating the SG (six times for 3 min each with a pulse ratio of 2 sec on / 2 sec off) and centrifuging the crude extract at 10,000 g for 15 min at 4°C. The supernatant was filtered on a 0,22 μm filter and transferred to clean tubes. Protein concentration was estimated with a Nanodrop (~0,75 mg/mL) and used as quality control before storage of SGE at −80°C.

### Cell infection

Human neuroblastoma SK-N-SH cells were maintained at 37°C in DMEM with 10% FBS. Cells were seeded in 6-well plates with 5.10^5^ cells/well (3 wells/condition) for 24h and infected with JEV genotype 3 strain RP-9 [5] or WNV strain IS98 [28] (produced on C6/36 cells) at a MOI of 1 in 1 mL of DMEM with 2% FBS, in presence or not of SGE (2 μL, equivalent of 2 SG). After 48h, the supernatant was removed from the 6-well plates.

For proteomic analysis, cells were resuspended in label free denaturation buffer (Tris 100mM pH8.0, urea 8M). Whole cell protein extract was obtained by sonication as described for the SGE. The supernatant was transferred to clean tubes, consistency between samples was confirmed by OD measurement with a Nanodrop and the samples were stored at -80°C. Each experiment was performed in biological triplicate.

For western blotting, cells were washed, scrapped in 1 mL of PBS, cells were centrifuged at 5000g and protein lysates were prepared by cell lysis in RIPA buffer (Bio Basic; catalog no. RB4476) containing protease inhibitors (Roche; catalog no. 11873580001).

### In-solution digestion of protein extracts

Reduction of disulfide bonds was performed in 5 mM dithiothreitol (DTT) for 30 min at room temperature; alkylation was performed in 20 mM iodoacetamide in the dark for 30 min at room temperature. Each sample was first digested by 500 ng of LysC (Promega, Madison, WI, USA) at 30°C for 3 hours. Solution was diluted in 100 mM ammonium bicarbonate (BA), until urea concentration was below 1.5 M. Each sample was then digested with 500 ng of trypsin (Promega, Madison, WI, USA) at 37°C overnight. Digestion was stopped by adding 1% formic acid (FA). Resulting peptides were purified using SPE C18 strategy and concentrated to almost dryness in 50% acetonitrile (ACN) 0.1% FA with a speedvac. Briefly, C18 phase (Sep-Pak, Waters) was activated in methanol, rinsed once in 80% ACN 0.1% FA, washed thrice in 0.1% FA. Resin was washed thrice in 0.1% FA, once in 2% ACN 0.1% FA. Peptides were eluted in 50% ACN 0.1% FA. Peptides in elution buffer were concentrated to almost dryness.

### Mass spectrometry analysis, database search, and protein identification

Digested peptides were analyzed by nano LC-MS/MS using an EASY-nLC 1000 (Thermo Fisher Scientific) coupled to a Q Exactive Orbitrap mass spectrometer. About 1 μg of each sample (dissolved in 0.1% FA) was loaded and separated at 250 nl.min-1 on a home-made C18 50 cm capillary column picotip silica emitter tip (75 μm diameter filled with 1.9 μm Reprosil-Pur Basic C18-HD resin, (Dr. Maisch GmbH, Ammerbuch-Entringen, Germany)) equilibrated in solvent A (0.1% FA). The peptides were eluted using a two slopes gradient of solvent B (0.1% FA in ACN) from 2% to 27% in 100 min and to 27% to 60% in 50 min at 250 nL/min flow rate (total length of the chromatographic run was 180 min). The Q Exactive (Thermo Fisher Scientific, Bremen) was operated in data-dependent acquisition mode with the XCalibur software 2.2 (Thermo Fisher Scientific, Bremen). Survey scan MS were acquired in the Orbitrap on the 300–1700 m/z range with the resolution set to a value of 70 000 at m/z = 400 in profile mode (AGC target at 1E6). The 5 most intense ions per survey scan were selected for HCD fragmentation (NCE 27), and the resulting fragments were analyzed in the Orbitrap at 17500 of resolution (m/z 400). Isolation of parent ion was fixed at 1.6 m/z and underfill ratio at 1%. Dynamic exclusion was employed within 45 sec.

Data were searched with the Andromeda search engine using MaxQuant (1.4.1.2 version) against the Human database from SwissProt and TrEMBL (2014.01.14, 88500 entries including 39715 from SwissProt), structural polyproteins of JEV and WNV, and the Culex pipiens database (2014.12.12, 130 entries, from Uniprot).

The following search parameters were applied: Carbamidomethylation of cysteines was set as a fixed modification. Oxidation of methionine and protein N-terminal acetylation were set as variable modifications. The mass tolerances in MS and MS/MS were set to 5 ppm for each, respectively. Maximum peptide charge was set to 7 and 5 amino acids were required as minimum peptide length. Two peptides were required for protein identification and quantitation. Peptides and proteins identified with an FDR lower than 0.1% were considered as valid identification.

Label free analysis was done by using the 'match between run' feature of MaxQuant (3 min time window). LFQ data were used to performed statistical analysis between conditions of infection.

### Statistical analysis of label free MS data

Statistical analysis of label free data was performed using the MS-Stat package on R environment [29]. Protein LFQ metrics were used for further statistical analysis. Proteins were declared significant with fold change higher than 1.5, and adjusted p-value below 5% of error.

### Bioinformatic analysis

For bioinformatic analysis, MS hit list was curated and annotated with both UniProt IDs and Entrez GeneIDs.

Gene ontology (GO) and Functional annotation clustering analysis was performed using DAVID v6.8 [30,31]. The degree of common genes between annotations was measured using Kappa statistics with a *similarity term overlap* of 4 and a *similarity threshold* of 0.7. Group of similar annotations were classified based on Kappa values and according to the following parameters: *initial group membership* of 2, *final group membership* of 3 and a *multiple linkage threshold* of 0.3. Finally, an *enrichment threshold* of 1.0 was used.

The interaction network for proteins of interest was obtained by using STRING v10 [32]. *High confidence* interactions (minimum score: 0.7) were determined using the following four sources: *text mining*, *experiments*, *databases* or *co-expression* and the network was solidified adding up to five *second shell interactors*–proteins which were not identified but connect identified proteins. Finally, the network was visualized with Cytoscape [33].

### Western blotting

Equal amounts of total proteins were loaded on a NuPAGE Novex 4 to 12% Bis-Tris protein gel (Life Technologies) and transferred to a PVDF membrane (Bio-Rad; catalog no. 170–4156). After blocking the membrane for 2h at room temperature in PBS-Tween (PBS-T) plus 5% milk, the blot was incubated overnight at 4°C with recommended dilutions of the primary antibodies. The membrane was then washed in PBS-T and incubated for 2h at RT in the presence of HRP-conjugated secondary antibodies. After washes in PBS-T, the membrane was incubated with the Pierce ECL Western blotting substrate (Thermo Scientific; catalog no. 32106) and protein bands were revealed using MyECL Imager machine (Thermofisher).

### Antibodies

Monoclonal antibody 4G2 anti-Flavivirus E protein was purchased from RD Biotech (Besançon, France). Rabbit polyclonal antibodies against IFIT3 (catalog no. GTX112442) and COL1A1 (catalog no. GTX112731) proteins were procured from GeneTex. Rabbit monoclonal antibody against PAM (catalog no. ab109175) and SSBP2 (catalog no. ab177944) were purchased from Abcam. Mice monoclonal antibody against actin beta was purchased from Thermo-Fischer (catalog no. MA1-140).

Horseradish peroxidase (HRP)-conjugated goat anti-mouse and anti-rabbit IgG antibodies were obtained from Bio-Rad Laboratories (catalog no. 170–6516 and 170–6515, respectively).

## Results

### Identification of differentially expressed proteins following JEV or WNV infection

**Fig 1:**
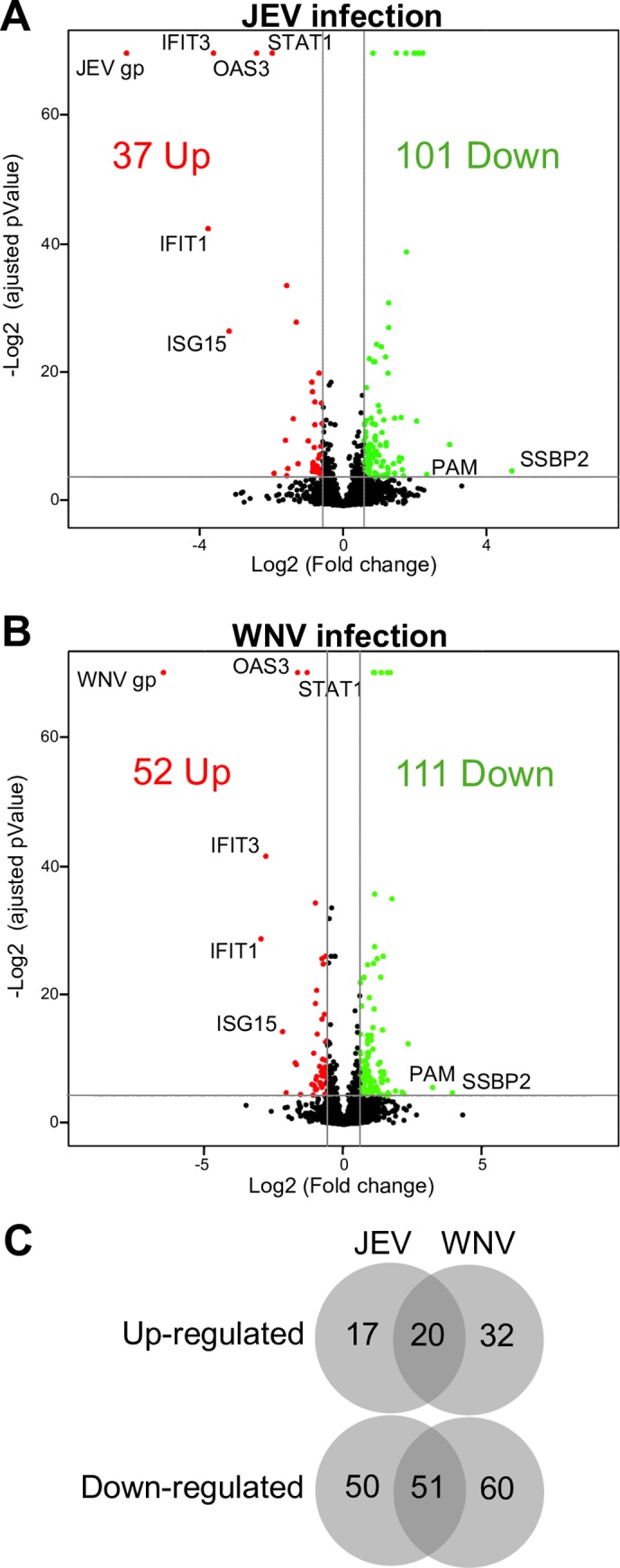
Comparison of human neuroblastoma proteome modulation during JEV or WNV infection. A-B. Volcano plots of protein expression in non-infected vs infected cells. SK-N-SH cells were infected for 48h with either JEV (A) or WNV (B) and proteins were extracted for label free quantitation by mass spectrometry. The results are representative of 3 independent experiments. Each spot represents a protein identified (black) and significantly down-regulated (green) or up-regulated (red) during viral infection. C. Venn diagram of the proteins up- and down-regulated in JEV (A) and WNV (B) infected cells.

In order to study the host proteome during neurotropic flavivirus infection in a human neuron model, SK-N-SH cells were infected with either JEV (genotype 3, strain RP-9) or WNV (strain IS-98) and, after 48h, proteins from whole-cell extracts were identified and quantified by label-free quantification mass spectrometry. Using the MaxQuant suite, a total of 3907 proteins were identified in all conditions (Mock, JEV- and WNV- infected cells). The genomic polyprotein of JEV and WNV were highly detected in the infected cells, confirming an efficient infection of the cells by both viruses (S2 Fig). Amongst the identified host proteins, a strong perturbation of the proteome was observed in response to both JEV and WNV infection (Fig 1).

In the case of JEV, the expression of 37 cellular proteins is up-regulated after 48h of infection (S1 Table) and that of 101 is down-regulated (S2 Table). Regarding WNV, the expression of 52 proteins is up-regulated (Table 1) and that of 111 is down-regulated (Table 2). While the median fold change (FC) is 1.8 for all conditions, the most up-regulated host proteins (Table 1) have a FC of 13.6 and 7.8, and the most down-regulated host proteins (Table 2) have a FC of 26 and 15 for JEV and WNV, respectively.

**Table 1:** Top 10 up-regulated proteins during JEV and/or WNV infection. Proteins are sorted according to the fold change in the JEV- (or secondly in WNV-) infected cells compared to the Mock. For complete list, see S1 Table.

Effect	Uniprot #	GeneID #	Gene symbol	Name	Clusters	JEV/Mock FC (pValue)	WNV/Mock FC (pValue)
Up-regulated during JEV infection	*P32886*	*1489713*	N/A	**JEV genomic polyprotein**	* *	*65*.*95 (0*.*00E+00)*	* *
	Q5EBM0-4	129607	CMPK2	UMP-CMP kinase 2, mitochondrial	Nucleotide-binding	3.82 (3.25E-02)	
	O60664	10226	PLIN3	Perilipin-3	Cell-cell adhesion	1.82 (1.81E-06)	
	P11047	3915	LAMC1	Laminin subunit gamma-1	Collagen metabolism, EGF-like domain, Extracellular matrix org., Glycoprotein	1.80 (5.28E-06)	
	Q9BXJ9	80155	NAA15	N-alpha-acetyltransferase 15, NatA auxiliary subunit	Tetratricopeptide repeat, Transcription regulation	1.78 (1.80E-02)	
	O00622	3491	CYR61	Protein CYR61	Glycoprotein, Nucleotide-binding	1.76 (1.82E-02)	
	Q9Y520-4	23215	PRRC2C	Protein PRRC2C		1.76 (1.16E-02)	
	Q9Y3Z3	25939	SAMHD1	Deoxynucleoside triphosphate triphosphohydrolase SAMHD1	Innate immunity	1.74 (1.58E-05)	
	F8VVL1	8562	DENR	Density-regulated protein		1.66 (2.90E-02)	
	Q9UNF0-2	11252	PACSIN2	Protein kinase C and casein kinase substrate in neurons protein 2	Cell-cell adhesion	1.66 (2.26E-02)	
Up-regulated during both JEV and WNV infection	P09914	3434	IFIT1	Interferon-induced protein with tetratricopeptide repeats 1	Innate immunity, Tetratricopeptide repeat	13.60 (1.34E-13)	7.838 (2.36E-09)
	O14879	3437	IFIT3	Interferon-induced protein with tetratricopeptide repeats 3	Innate immunity, Tetratricopeptide repeat	12.31 (0.00E+00)	6.944 (3.20E-13)
	P05161	9636	ISG15	Ubiquitin-like protein ISG15	Anchored protein, Innate immunity	9.08 (7.93E-09)	4.59 (4.85E-05)
	Q9Y6K5	4940	OAS3	2'-5'-oligoadenylate synthase 3	Innate immunity, Nucleotide-binding, Transmembrane protein	5.32 (0.00E+00)	3.17 (0.00E+00)
	P42224	6772	STAT1	Signal transducer and activator of transcription 1-alpha/beta	Cell-cell adhesion, Innate immunity, Transcription regulation	3.96 (0.00E+00)	2.49 (0.00E+00)
	Q9BQE5	23780	APOL2	Apolipoprotein L2	Transmembrane protein	3.04 (9.67E-04)	2.13 (4.43E-02)
	Q13325	24138	IFIT5	Interferon-induced protein with tetratricopeptide repeats 5	Innate immunity, Tetratricopeptide repeat, Transmembrane protein	2.98 (5.96E-11)	1.93 (6.50E-05)
	Q8WUP2-3	54751	FBLIM1	Filamin-binding LIM protein 1		2.96 (4.13E-02)	2.94 (4.26E-02)
	Q5K651	54809	SAMD9	Sterile alpha motif domain-containing protein 9		2.61 (9.24E-05)	2.11 (4.97E-04)
	Q9NUQ6-2	26010	SPATS2L	SPATS2-like protein		2.47 (2.95E-09)	2.02 (2.37E-06)
Up-regulated during WNV infection	*P06935*	*912267*	N/A	**WNV genomic polyprotein**	* *	* *	*271*.*78 (3*.*88E-04)*
	Q6GMV3	391356	PTRHD1	Putative peptidyl-tRNA hydrolase PTRHD1			4.22 (3.58E-02)
	Q5QNY5	5824	PEX19	Peroxisomal biogenesis factor 19	Transmembrane		3.35 (1.43E-03)
	Q96C90	26472	PP1R14B	Protein phosphatase 1 regulatory subunit 14B			2.21 (1.43E-02)
	P0DMV8/9	3303	HSPA1A	Heat shock 70 kDa protein 1A	Cell-cell adhesion, Nucleotide-binding		2.03 (4.86E-11)
	Q9Y6G9	51143	YNC1LI1	Cytoplasmic dynein 1 light intermediate chain 1	Nucleotide-binding		2.01 (2.58E-02)
	Q8TDX7	140609	NEK7	Serine/threonine-protein kinase Nek7	Kinase, Nucleotide-binding		1.99 (8.66E-03)
	Q9BUF5	84617	TUBB6	Tubulin beta-6 chain	Nucleotide-binding		1.96 (2.40E-02)
	O95817	9531	BAG3	BAG family molecular chaperone regulator 3	Cell-cell adhesion		1.96 (6.14E-03)
	O00592-2	5420	PODXL	Podocalyxin	Glycoprotein, Transmembrane		1.77 (1.43E-02)

**Table 2:** Top 10 down-regulated proteins during JEV and/or WNV infection. Proteins are sorted according to the fold change in the JEV- (or secondly in WNV-) infected cells compared to the Mock. For complete list, see S2 Table.

Effect	Uniprot #	GeneID #	Gene symbol	Name	Clusters	JEV/Mock FC (pValue)	WNV/Mock FC (pValue)
Down-regulated during JEV infection	Q99797	4285	MIPEP	Mitochondrial intermediate peptidase	Metalloprotease	7.84 (1.53E-03)	
	P21810	633	BGN	Biglycan	Extracellular matrix org., Glycoprotein, Leucine-rich repeat	3.21 (4.36E-02)	
	Q8WZ42-5	7273	TTN	Titin	Immunoglobulin domain, Nucleotide-binding, Tetratricopeptide repeat	3.12 (2.25E-02)	
	O14657	27348	TOR1B	Torsin-1B	Glycoprotein, Nucleotide-binding	3.09 (6.62E-03)	
	P55083	4239	MFAP4	Microfibril-associated glycoprotein 4	Extracellular matrix org., Glycoprotein	2.95 (6.07E-03)	
	O75063	9917	FAM20B	Glycosaminoglycan xylosylkinase	Glycoprotein, Nucleotide-binding, Transmembrane protein	2.70 (3.24E-02)	
	Q9P032	29078	NDUFAF4	NADH dehydrogenase [ubiquinone] 1 alpha subcomplex assembly factor 4		2.37 (2.00E-02)	
	Q13641	7162	TPBG	Trophoblast glycoprotein	Glycoprotein, Leucine-rich repeat, Transmembrane protein	2.36 (4.55E-02)	
	Q86UV5-2	84196	USP48	Ubiquitin carboxyl-terminal hydrolase 48		2.24 (3.92E-02)	
	P63218	2787	GNG5	Guanine nucleotide-binding protein G(I)/G(S)/G(O) subunit gamma-5	Anchored protein, Collagen metabolism	2.22 (4.35E-02)	
Down-regulated during both JEV and WNV infection	P81877-2	23635	SSBP2	Single-stranded DNA-binding protein 2		26.22 (2.60E-02)	15,05 (3.46E-02)
	P19021-2	5066	PAM	Peptidyl-glycine alpha-amidating monooxygenase	Glycoprotein, Transmembrane protein	5.05 (3.74E-02)	9,23 (2.02E-02)
	P02452	1277	COL1A1	Collagen alpha-1(I) chain	Extracellular matrix org., Collagen metabolism, Glycoprotein, Transmembrane protein	4.65 (0.00E+00)	2,96 (0.00E+00)
	P08123	1278	COL1A2	Collagen alpha-2(I) chain	Extracellular matrix org., Collagen metabolism, Glycoprotein	4.29 (0.00E+00)	2,16 (5.21E-09)
	P02461	1281	COL3A1	Collagen alpha-1(III) chain	Extracellular matrix org., Collagen metabolism, Glycoprotein	4.28 (0.00E+00)	2,16 (1.89E-11)
	Q9H3M7	10628	TXNIP	Thioredoxin-interacting protein	Transcription regulation	4.13 (1.20E-04)	4,98 (1.82E-04)
	Q96CG8	115908	CTHRC1	Collagen triple helix repeat-containing protein 1	Extracellular matrix org., Collagen metabolism, Glycoprotein	3.98 (0.00E+00)	2,54 (0.00E+00)
	O95864	9415	FADS2	Fatty acid desaturase 2	Transmembrane protein	3.41 (1.60E-12)	3,36 (3.20E-11)
	Q15113	5118	PCOLCE	Procollagen C-endopeptidase enhancer 1	Extracellular matrix org., Collagen metabolism, Glycoprotein	3.35 (0.00E+00)	1,81 (3.60E-08)
	C9JEZ4	10602	CDC42EP3	Cdc42 effector protein 3	Transmembrane protein	3.09 (8.20E-05)	2,26 (1.88E-02)
Down-regulated during WNV infection	P33908	4121	MAN1A1	Mannosyl-oligosaccharide 1,2-alpha-mannosidase IA	Glycoprotein, Transmembrane		4.52 (4.01E-02)
	B4DWB0	80224	NUBPL	Iron-sulfur protein NUBPL	Glycoprotein, Nucleotide-binding		4.30 (3.39E-02)
	Q8N129	245812	CNPY4	Protein canopy homolog 4	Glycoprotein		3.65 (2.81E-02)
	H3BQR0	10073	SNUPN	Snurportin-1			3.10 (4.33E-02)
	Q16706	4124	MAN2A1	Alpha-mannosidase 2	Glycoprotein, Transmembrane		2.98 (3.58E-02)
	Q9Y294	25842	ASF1A	Histone chaperone ASF1A	Transcription regulation		2.93 (4.58E-02)
	Q9NRX1	56902	PNO1	RNA-binding protein PNO1			2.70 (4.97E-03)
	P07711	1514	CTSL	Cathepsin L1	Collagen metabolism, Glycoprotein, Lysosome		2.63 (4.66E-02)
	P38571-2	3988	LIPA	Lysosomal acid lipase/cholesteryl ester hydrolase	Glycoprotein, Lysosome		2.58 (1.19E-02)
	O00754-2	4125	MAN2B1	Lysosomal alpha-mannosidase	Glycoprotein, Lysosome		2.53 (6.58E-03)

Interestingly, the expression of 20 proteins is consistently up-regulated during infection by JEV and WNV (Fig 1C), including innate immunity-related proteins such as IFIT-1, IFIT-3, ISG15, OAS and STAT1 which are amongst the most up-regulated proteins for both JEV and WNV (FC ranging from 2.9 to 13.6 for JEV and from 1.9 to 7.8 for WNV, Table 1). Conversely, 51 proteins are consistently down-regulated by both JEV and WNV (Fig 1C), of which proteins related to collagen metabolism were the most prominent (11 out of 51, S2 Table). Further, two common proteins between JEV and WNV were amongst the most down-regulated ones: SSBP2 (Single-stranded DNA-binding protein 2) and PAM (Peptidyl-glycine α-amidating monooxygenase). SSBP2 was also the protein most down-regulated by both viruses (FC of 26 and 15, respectively) (Table 2).

### Global host proteome response to JEV or WNV infection

**Fig 2:**
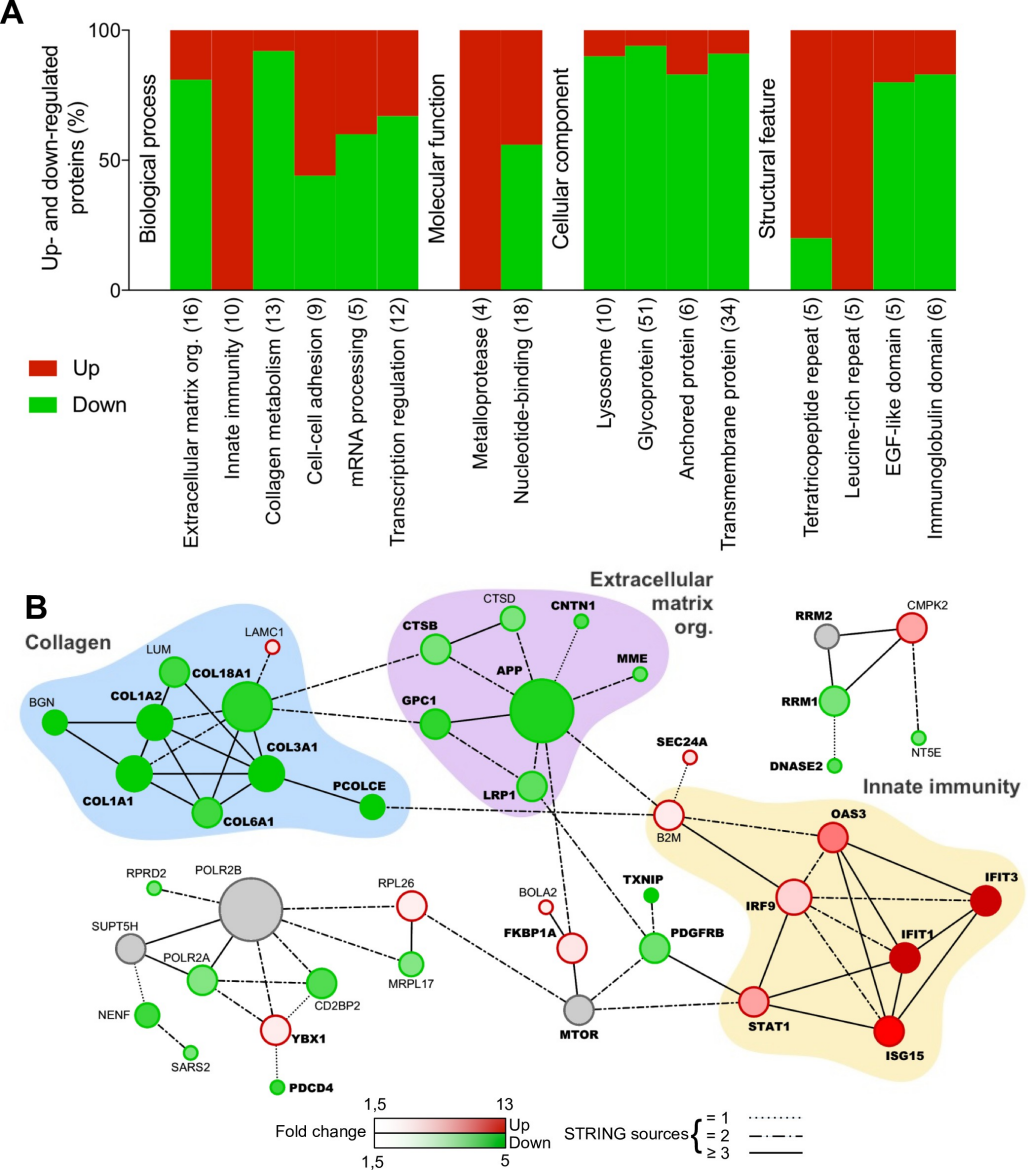
Functional clustering and network analysis of proteins modulated during JEV infection. A. Percentage of proteins up(red)- and down(green)-regulated in functional groups. Modulated proteins were clustered into functional groups using DAVID v6.8 (detailed in S1 Fig). Functional groups are organized in four domains: Biological process, Molecular function, Cellular component or Structural feature and according to their enrichment score from the left to the right. Total number of proteins associated to each group is noted between brackets. B. Networks of up(red)- and down(green)-regulated proteins. Protein-protein interactions (PPI) networks were determined with STRING v10 and visualized with Cytoscape. Proteins regulated in common with WNV are highlighted in bold. Node size is relative to the number of edges. Grey nodes correspond to second shell proteins linking identified proteins. Edges are determined according to the number of sources (text mining, experiments, databases or co-expression) supporting the link between proteins.

**Fig 3:**
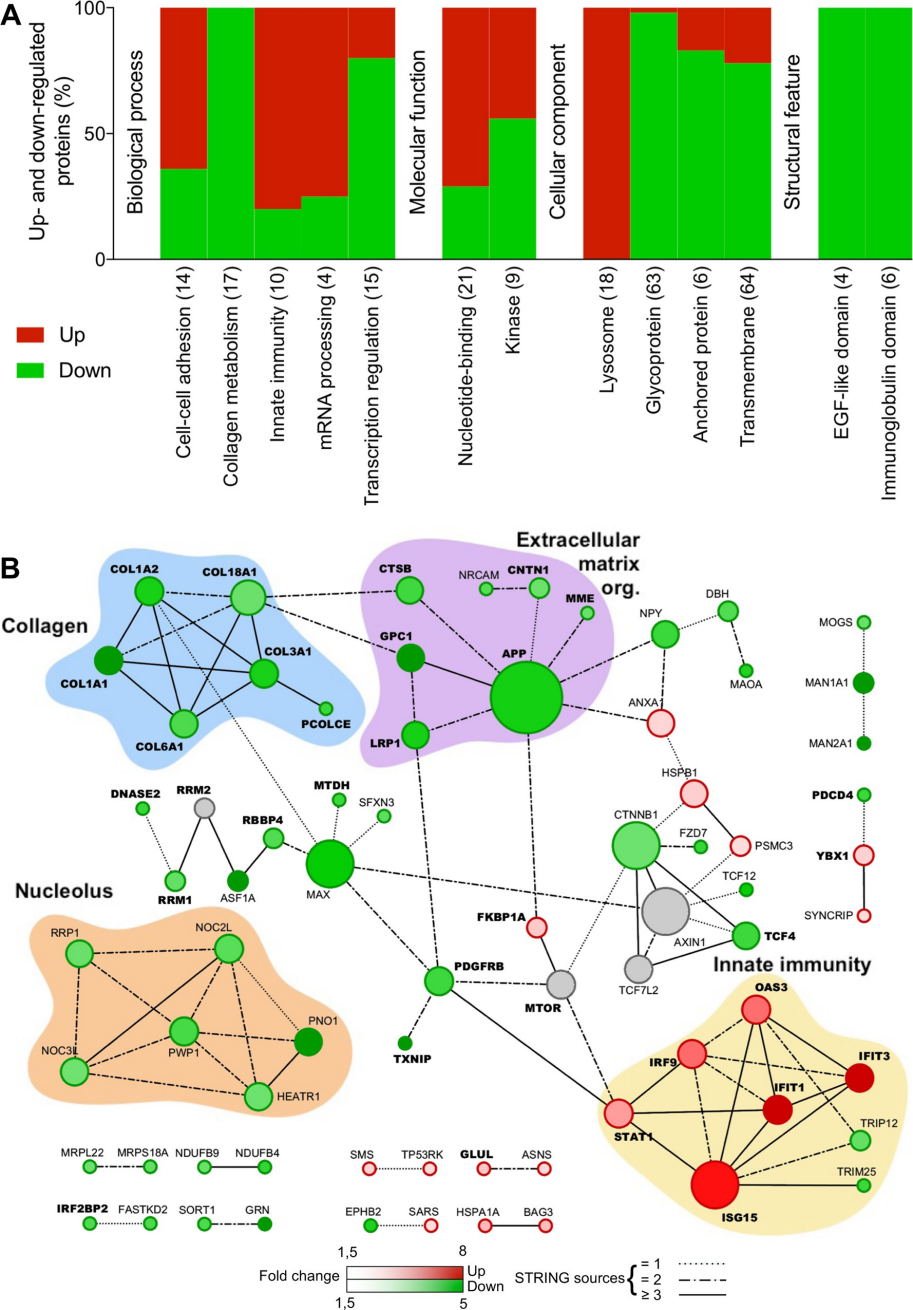
Functional clustering and network analysis of proteins modulated during WNV infection. A. Percentage of proteins up(red)- and down(green)-regulated in functional groups. Modulated proteins were clustered into functional groups using DAVID (detailed in S2 Table). Functional groups are organized in four domains: Biological process, Molecular function, Cellular component or Structural feature and according to their enrichment score from left to right. Total number of proteins associated to each group is noted between brackets. B. Networks of up(red)- and down(green)-regulated proteins. PPI networks were determined with STRING and visualized with Cytoscape. Proteins regulated in common with JEV are highlighted in bold. Node size is relative to the number of edges. Grey nodes correspond to second shell proteins linking identified proteins. Edges are determined according to the number of sources (text mining, experiments, databases or co-expression) supporting the link between proteins.

Functionally related groups of proteins regulated during JEV or WNV neuroblastoma cell infection were identified using DAVID functional annotation clustering tool (Figs 2A and 3A and S3 and S4 Tables). About 72% of the proteins modulated during JEV infection were assigned to 16 functional groups (Fig 2A) and 78% of the proteins modulated during WNV infection were assigned to 13 functional groups (Fig 3A). Protein-protein interaction networks were also established using STRING, highlighting interactions within 32% and 45% of the proteins modulated during JEV (Fig 2B) or WNV (Fig 3B) infection, respectively.

In response to JEV infection, a functional cluster of 10 proteins involved in immunity was strictly up-regulated (FC from 13.6 to 1.7): IFIT1, IFIT3, ISG15, OAS3, STAT1, IFIT5, IRF9, SAMHD1, B2M, ZC3HAV1 (Fig 2A, Table 1 and S1 Table) of which 7 proteins also formed a cluster of interacting proteins: IFIT1, IFIT3, ISG15, OAS3, STAT1, IRF9 and B2M (Fig 2B). The tetratricopeptide repeat cluster (including proteins from the IFIT family) was the only other cluster substantially up-regulated (4/5). Conversely, clusters of proteins part of the extracellular matrix organization (13/16) and related collagen metabolism (12/13) were the most down-regulated biological processes (Fig 2A). Both functional annotation clusters were also confirmed in the network analysis (Fig 2B). About half of the down-regulated proteins are glycoproteins (48/101) and one third are transmembrane or anchored proteins (36/101) while they are under-represented amongst up-regulated proteins (Fig 2A). A cluster of proteins associated to the lysosomes was down-regulated (9/10) and several metalloproteases or proteins featuring leucine-rich repeats, EGF-like domains or immunoglobulin domains were also mostly down-regulated (Fig 2A). Finally, mRNA processing and transcription regulation clusters and the nucleotide-binding cluster were either up- or down-regulated during JEV infection (Fig 2A). Fig 2B highlights a small group of proteins modulated during JEV infection involved in transcription regulation and mRNA processing (RPRD2, POLR2A, YBX1, CD2BP2) and revolving around subunits of DNA-dependent RNA polymerase II.

In response to WNV infection, similar clusters and networks of modulated proteins were observed (Fig 3). Proteins involved in immune response such as IFIT1, IFIT3, ISG15, OAS3, STAT1, IFIT5 or IRF9 were also up-regulated together with ANXA1 (Table 1). However, two proteins, ERAP2 involved in antigen processing and TRIM25 involved in IFN triggering, were both down-regulated (Fig 3, Table 2). The down-regulation of extracellular matrix organization was only identified using network analysis (Fig 3B), and while not being a key characteristic as for JEV infection (Fig 2), it remains an important feature of WNV infection. Moreover, the cluster of 17 proteins involved in collagen metabolism was also strictly down-regulated (Fig 3A) while 9/14 proteins involved in cell-cell adhesion were down-regulated during WNV infection. About half of the down-regulated proteins were glycoproteins (62/111) and anchored or transmembrane proteins (56/111) while they were under-represented amongst up-regulated proteins (Fig 3A). A cluster of 18 proteins associated to lysosomes was strictly down-regulated and several proteins featuring EGF-like domains or immunoglobulin domains were also mostly down-regulated while a cluster of 9 protein kinases was either up- or down-regulated (Fig 3A). Finally, mRNA processing (3/4) and nucleotide-binding (15/21) clusters were noticeably up-regulated during WNV infection (Fig 3A) which can be correlated to a network of down-regulated proteins linked to the nucleolus (Fig 3B), while conversely transcription regulation cluster was substantially up-regulated (12/15).

**Fig 4:**
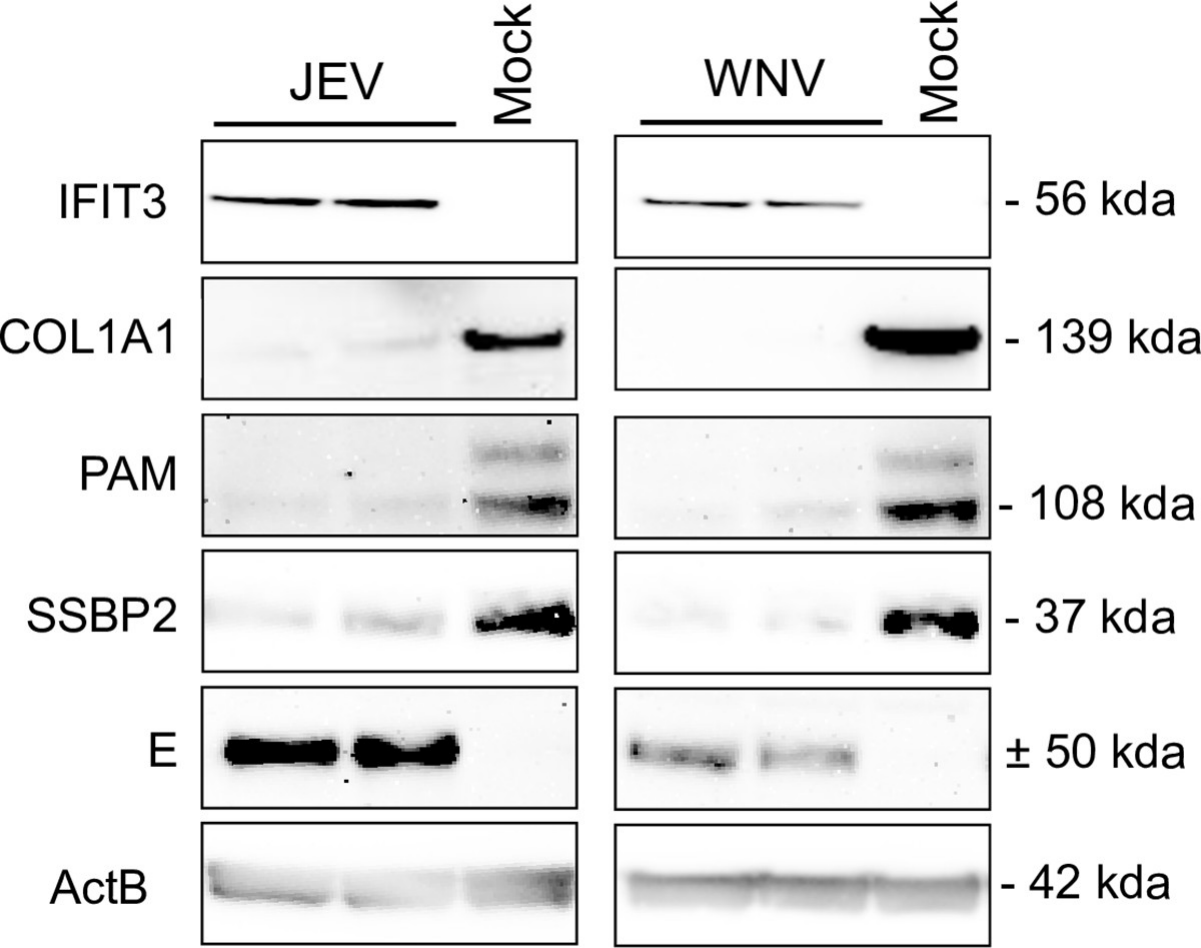
JEV and WNV inhibit collagen, PAM and SSBP2 expression in a neuron model. SK-N-SH cells were infected with JEV or WNV at a MOI of 1 and proteins were extracted for western blot analysis 48hpi. Two independent experiments are displayed on the blot.

Overall, functional annotation clustering and protein-protein interaction networks show strong similarities between JEV and WNV infection of neuroblastoma cells. In order to confirm our findings, we further selected two hits representative of two of the most important clusters: IFIT3 for the innate immunity and COL1A1 for collagen organization, as well as the two hits associated with the strongest downregulation: SSBP2 and PAM (Fig 4). As expected, IFIT3 was expressed in response to JEV or WNV infection while COL1A1, SSPB2 and PAM expression was greatly reduced. It is worth noting that no cytopathic effect was observed despite the reduction of collagen expression (data not shown).

### Role of mosquito saliva in a neuron model of infection

As saliva from mosquitoes is known to enhance *Flavivirus* infection [19,20], including WNV [21,23], we tested its potential effect on human neuroblastoma cell infection by JEV or WNV and on the proteome of the infected cells. For this purpose, we infected SK-N-SH cells with JEV or WNV in the presence of mosquito salivary gland extracts (SGE) and, after 48h, proteins from whole-cell extracts were identified and quantified by label-free quantification mass spectrometry. We identified only one *Cx pipiens* protein in our samples among the 130 referenced in our MS data bank (Q15G69, ß-tubulin). No effect of SGE on viral replication could be detected as shown by viral titration and genomic polyprotein quantification (S2 Fig).

**Fig 5:**
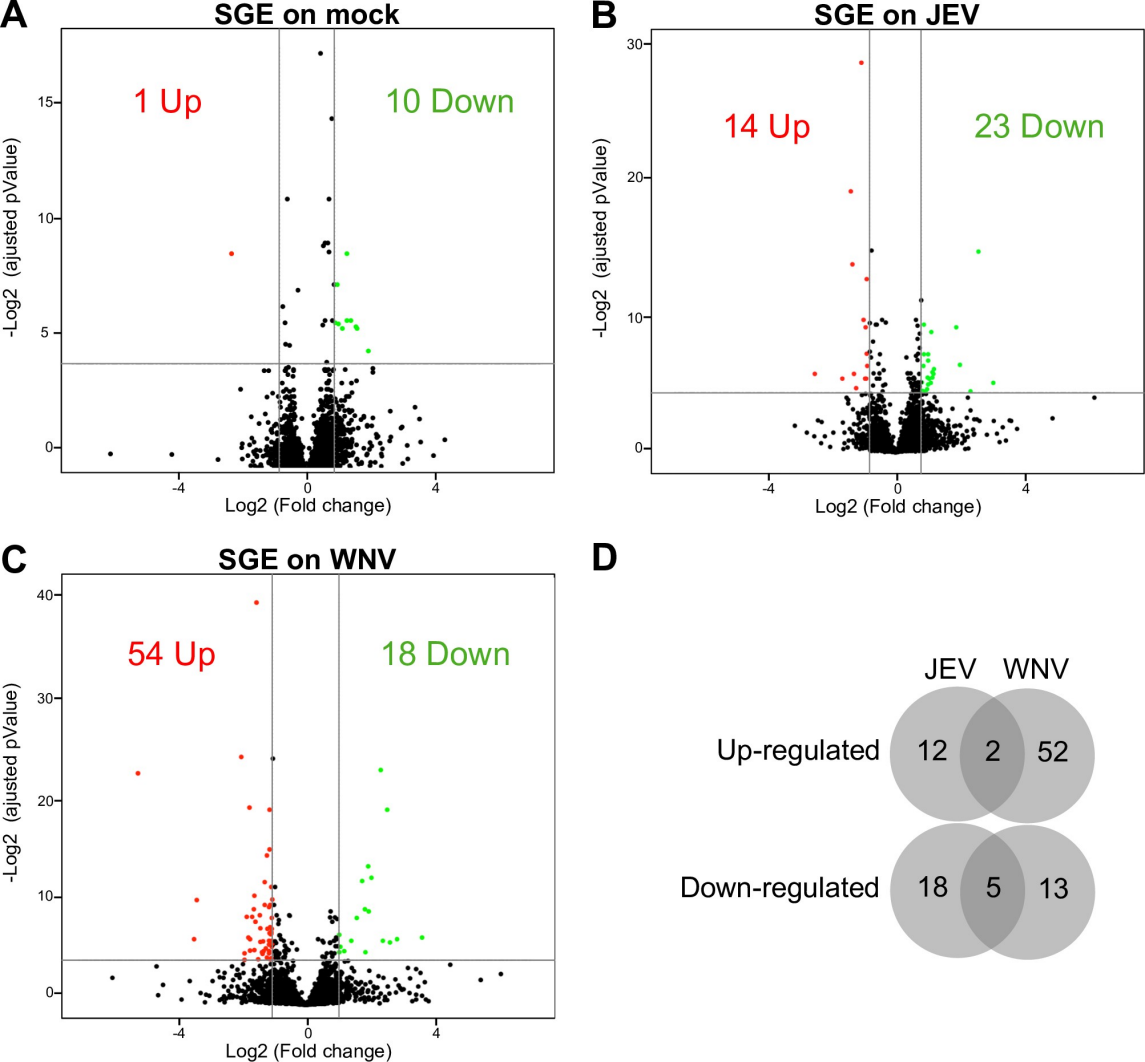
Comparison of modulation of the human neuroblastoma proteome during JEV or WNV infection in the presence of mosquito SGE. Volcano plots of protein expression in non-infected vs infected cells. After 48h, proteins from mock (A), JEV (B) or WNV (C) infected SK-N-SH cells were extracted for label free quantification by mass spectrometry. The results are representative of 3 independent experiments. Each spot represents a protein identified (black) and significantly down-regulated (blue) or up-regulated (red) during viral infection.

In the absence of virus, mosquito SGE had little effect on the cells (Fig 5A). We observed only one up-regulated protein, ZRANB2 (FC of 3.0), a zinc-finger protein involved in RNA splicing, and 10 down-regulated proteins with a FC ranging from 1.51 to 2.45 (Table 3). Notably, out of all down-regulated proteins, 4 were involved in nuclear transport. On the other hand, mosquito SGE had a substantially stronger effect on the proteome of JEV (Fig 5B) or WNV (Fig 5C) infected cells. Interestingly, only a few proteins were similarly regulated for the 2 viruses: 2 up-regulated proteins, OSBPL8, a lipid transporter and HIST1H1B, a histone protein (Table 4) and 5 down-regulated proteins (Table 5), S100A6, a calcium sensor, NACA and EIF4H, two translation co-factors, COPS8, a component of the signalosome complex and LGALS1, a lectin.

**Table 3:** Effect of SGE on non infected human neuroblastoma cells.

Effect	Uniprot #	GeneID #	Gene symbol	Name	Fold change (adjusted pValue)	
Up-regulated in presence of SGE	O95218-2	9406	ZRANB2	zinc finger RANBP2-type containing 2	3.04 (2,33E-03)	
Down-regulated in presence of SGE	Q5QNY5	5824	PEX19	peroxisomal biogenesis factor 19	2.45 (3,45E-02)	
	E9PJ95	29099	COMMD9	COMM domain containing 9	2.08 (1,87E-02)	
	E7EQI7	9897	KIAA0196	KIAA0196	2.04 (1,77E-02)	
	Q9NRG7-2	56948	SDR39U1	short chain dehydrogenase/reductase family 39U member 1	1.91 (1,49E-02)	
	Q66K74	55201	MAP1S	microtubule associated protein 1S	1.79 (1,49E-02)	
	O43592	11260	XPOT	exportin for tRNA	1.79 (2,33E-03)	*
	Q9BZH6	55717	WDR11	WD repeat domain 11	1.68 (1,87E-02)	*
	O60443	1687	DFNA5	deafness associated tumor suppressor	1.58 (1,65E-02)	
	Q9HAV4	57510	XPO5	exportin 5	1.56 (5,53E-03)	*
	O95373	10527	IPO7	importin 7	1.51 (1,58E-02)	*
* involved in nuclear transport							

**Table 4:** Top 10 proteins up-regulated by SGE in JEV and/or WNV infected cells. Proteins are sorted according to the fold change in the JEV- (or secondly in WNV-) infected cells treated or not with SGE. For complete list, see S5 Table.

Effect	Protein #	Gene ID #	Gene	Gene name	JEV infected FC (adj. pValue)	WNV infected FC (adj. pValue)
Up-regulated proteins during JEV infection	Q15388	9804	TOMM20	translocase of outer mitochondrial membrane 20	3.52 (1,94E-02)	
	P16403	3006	HIST1H1C	histone cluster 1 H1 family member c	2.28 (2,48E-02)	
	P07305	3005	H1F0	H1 histone family member 0	1.96 (7,76E-05)	
	P53999	10923	SUB1	SUB1 homolog transcriptional regulator	1.92 (1,93E-02)	
	Q5TAQ0	84300	UQCC2	ubiquinol-cytochrome c reductase complex assembly factor 2	1.85 (4,01E-02)	
	Q9NR30	9188	DDX21	DExD-box helicase 21	1.70 (3,02E-09)	
	P11387	7150	TOP1	topoisomerase DNA I	1.64 (1,27E-03)	
	O15446	10849	CD3EAP	CD3e molecule associated protein	1.60 (2,48E-02)	
	Q9UIG0-2	9031	BAZ1B	bromodomain adjacent to zinc finger domain 1B	1.59 (1,85E-03)	
	P11388	7153	TOP2A	topoisomerase DNA II alpha	1.57 (2,48E-02)	
Up-regulated proteins during JEV and WNV infection	Q9BZF1-3	114882	OSBPL8	oxysterol binding protein like 8	1.57 (1,65E-04)	1.55 (2,83E-05)
	P16401	3009	HIST1H1B	histone cluster 1 H1 family member b	2.01 (1,98E-06)	1.50 (2,85E-03)
Up-regulated proteins during WNV infection	P60468	10952	SEC61B	Sec61 translocon beta subunit		7.71 (1,61E-07)
	H0YI58	51290	ERGIC2	ERGIC and golgi 2		3.90 (1,23E-02)
	Q9P0L0	9218	VAPA	VAMP associated protein A		3.78 (8,72E-04)
	P07099	2052	EPHX1	epoxide hydrolase 1		2.18 (5,44E-08)
	O15427	9123	SLC16A3	solute carrier family 16 member 3		2.10 (4,83E-02)
	Q13619	8451	CUL4A	cullin 4A		2.09 (3,11E-02)
	O00264	10857	PGRMC1	progesterone receptor membrane component 1		2.04 (2,72E-03)
	Q9BT22	56052	ALG1	ALG1, chitobiosyldiphosphodolichol beta-mannosyltransferase		2.01 (1,08E-02)
	P04844	6185	RPN2	ribophorin II		1.98 (1,66E-06)
	Q9HC07	55858	TMEM165	transmembrane protein 165		1.97 (2,59E-02)

**Table 5:** Top 10 proteins down-regulated by SGE in JEV and/or WNV infected cells. Proteins are sorted according to the fold change in the JEV- (or secondly in WNV-) infected cells treated or not with SGE. For complete list, see S6 Table.

Effect	Uniprot #	GeneID #	Gene symbol	Name	JEV infected FC (adj. pValue)	WNV infected FC (adj. pValue)
Down-regulated proteins during JEV infection	Q99797	4285	MIPEP	mitochondrial intermediate peptidase	4.61 (3,06E-02)	
	Q9Y6W5-2	10163	WASF2	WAS protein family member 2	3.67 (4,08E-05)	
	E7ESU0	10869	USP19	ubiquitin specific peptidase 19	3.24 (4,61E-02)	
	Q9BZX2	7371	UCK2	uridine-cytidine kinase 2	2.58 (1,85E-03)	
	O95400	10421	CD2BP2	CD2 cytoplasmic tail binding protein 2	1.82 (1,53E-02)	
	P00338	3939	LDHA	lactate dehydrogenase A	1.77 (2,33E-02)	
	Q9Y316	51072	MEMO1	mediator of cell motility 1	1.75 (2,34E-03)	
	Q9H3M7	10628	TXNIP	thioredoxin interacting protein	1.74 (3,06E-02)	
	Q9NRG7-2	56948	SDR39U1	short chain dehydrogenase/reductase family 39U member 1	1.69 (2,48E-02)	
	Q9NPQ8-4	60626	RIC8A	RIC8 guanine nucleotide exchange factor A	1.66 (3,29E-02)	
Down-regulated proteins during JEV and WNV infection	R4GN98	6277	S100A6	S100 calcium binding protein A6	2.73 (1,22E-02)	2.78 (1,49E-02)
	F8VZJ2	4666	NACA	nascent polypeptide-associated complex alpha subunit	1.80 (1,94E-02)	2.15 (1,88E-03)
	Q15056-2	7458	EIF4H	eukaryotic translation initiation factor 4H	1.78 (1,80E-02)	2.05 (1,60E-03)
	E9PGT6	10920	COPS8	COP9 signalosome subunit 8	1.67 (9,82E-03)	2.22 (1,93E-04)
	P09382	3956	LGALS1	galectin 1	1.66 (7,22E-03)	1.98 (2,36E-04)
Down-regulated proteins during WNV infection	Q9P0S9	51522	TMEM14C	transmembrane protein 14C		4.12 (1,10E-02)
	Q9NRX1	56902	PNO1	partner of NOB1 homolog		3.03 (1,23E-02)
	P61758	7411	VBP1	VHL binding protein 1		2.69 (1,93E-06)
	P49366	1725	DHPS	deoxyhypusine synthase		2.56 (1,36E-02)
	Q99497	11315	PARK7	Parkinsonism associated deglycase		2.49 (1,32E-07)
	Q04837	6742	SSBP1	single stranded DNA binding protein 1		2.14 (8,85E-05)
	O00499-9	274	BIN1	bridging integrator 1		2.06 (2,90E-02)
	O00743	5537	PPP6C	protein phosphatase 6 catalytic subunit		1.85 (2,85E-03)
	Q9Y3B8	25996	REXO2	RNA exonuclease 2		1.73 (1,36E-02)
	P46777	6125	RPL5	ribosomal protein L5		1.60 (2,73E-02)

**Fig 6:**
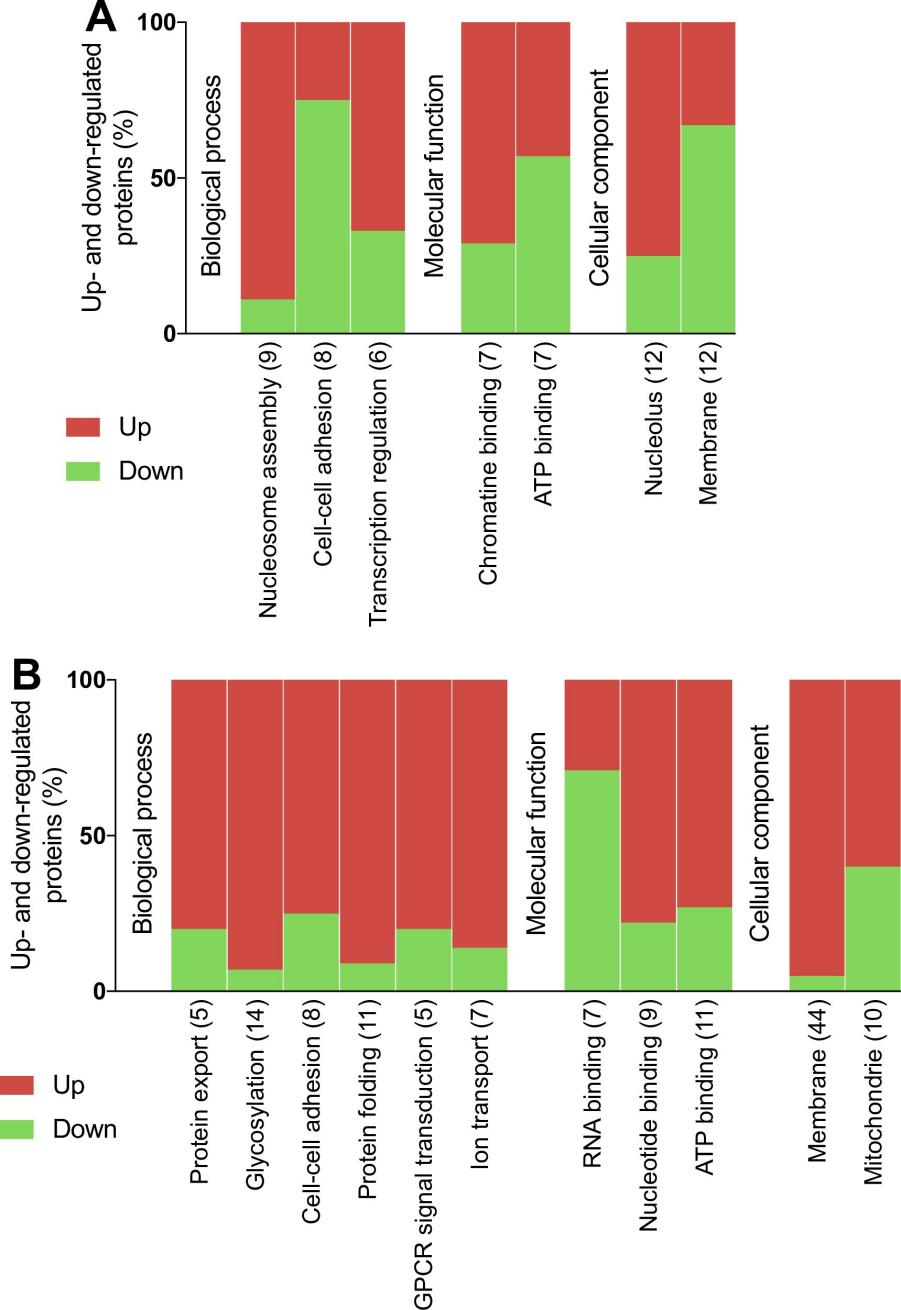
Functional clustering analysis of proteins modulated by mosquito SGE during JEV or WNV infection. Percentage of proteins up(red)- and down(green)-regulated in functional groups after JEV (A) or WNV (B) infection in the presence of mosquito SGE. Modulated proteins were clustered into functional groups using DAVID. Functional groups are organized in four domains: Biological process, Molecular function, Cellular component or Structural feature and according to their enrichment score from left to right. Total number of proteins associated to each group is noted between brackets.

When comparing JEV infected cells in presence or not of mosquito SGE (Fig 5B), we found that SGE induced a change in protein expression: either an increased expression with a FC ranging from 1.5 to 3.53 for 14 protein or a decreased expression with a FC ranging from 1.5 to 4.61 for 23 proteins. In a functional annotation clustering analysis (Fig 6A), mosquito SGE was shown to mostly up-regulate proteins involved in nucleosome assembly (9/12), transcription regulation (4/6), associated with the nucleolus (8/9) or with chromatin binding functions (5/7). Conversely, SGE was shown to mostly down-regulate membrane-associated proteins (8/12) involved in cell-cell adhesion (6/8) (Fig 6A).

In comparison with WNV infected cells in the presence or not of mosquito SGE (Fig 5C), 54 proteins were up-regulated in presence of SGE while 18 proteins were down-regulated (with a FC ranging from 1.5 to 7.71 and 1.5 to 4.12, respectively). In the case of WNV, functional annotation clustering (Fig 6B) showed a strong up-regulation of proteins involved in protein export (4/5) and protein folding (10/11), glycosylation (13/14) and cell-cell adhesion (6/8), as well as GPCR signal transduction (4/5) and ion transmembrane export (6/7). Many up-regulated proteins are located at the membrane (61% of all modulated proteins) or in the mitochondria (6/10) and exhibit ATP (8/11) or nucleotide (7/9) binding functions. Only RNA-binding proteins were substantially down-regulated by the presence of SGE (7/9).

Overall, mosquito SGE seems to have a strong impact on the proteome of human neuroblastoma cells infected by JEV or WNV. It is worth to note that the number of hits was much greater for the proteome of JEV or WNV infected cells in presence of SGE, as compared to that in the absence of SGE (166 up- and 174 down-regulated for JEV, 147 up- and 247 down-regulated for WNV, see S9 and S10 Tables). Resulting protein networks were considerably wider (S3 and S4 Figs), further confirming the influence of mosquito saliva on the response of a human neuron model to JEV or WNV infection.

## Discussion

Viral infections of the CNS still represent a challenge for modern medicine as they are associated with a high mortality rate [34]. To shed a new light on the infection of the CNS by two major neurotropic flaviviruses, we designed a comparative quantitative proteomic study of JEV or WNV infected human neuroblastoma cells. The quantification of viral genomic polyproteins and host proteins supports the view that the higher replication rate of WNV (IS98) is associated with a better control of the immune response compared to JEV (RP9) [35,36]. This variation in immune response control could correlate with the down-regulation of TRIM25 which was described to be essential to establish an IFN response to WNV infection [37]. Moreover, antigen processing is impaired in WNV infected fibroblasts [38], possibly in relation to the down-regulation of ERAP2.

Overall, neuroblastoma proteome profiles observed during JEV or WNV infection in our study show strong similarities in the way neurotropic flaviviruses disturb the host cell. A substantial number of proteins together with several functional clusters are similarly up- or down-regulated during JEV and WNV infections. This findings could correlate with the many similarities observed between the CNS diseases induced by JEV and WNV [39][40].

Surprisingly, our study corroborates only partially previous works on WNV- or JEV- infected cell proteome. We confirmed induction of the immune response by JEV previously observed in HeLa cells [8], as well as up-regulation of SAMD9 not only by JEV but also by WNV. From all the pathways and cellular factors identified in a study performed on mouse brain and neuroblastoma [9], only ERP29 was modulated in our work (S2 Table). However, while it was up-regulated in mouse cells, we observed a slight down-regulation in our human cells (FC: 1.66). Regarding WNV, our results on the immune response pathway and STAT1 factor corroborated that of [10] in Vero cells and of [11] in the mouse brain. Finally, GSK3B, PNKP and RB1, which were suggested as targets to control the neuroinflammation in response to WNV infection in the glioblastoma cell line U251 [12], were not modulated in our study. Altogether, these discrepancies may stem from the choice of cellular and animal model used to perform proteomic studies on flaviviruses [41] or possible technical limitations in the resolution of 2D-DIGE and labeled MS compared to whole-cell extract and label free MS [42].

As for the microarray-based study comparing JEV and WNV in mouse brain [13], we were able to confirm expression modulation of several cellular functions and notably up-regulation of the immune response: IFIT1, OAS1, STAT1 and IRF9 as well as IFIT3 which in our study was up-regulated by both JEV and WNV. Interestingly, modulation of tRNA charging, which was the only function determined as specific to flaviviruses [13], was also observed here. However, only SARS was up-regulated by WNV in our study, together with PTRHD1 which was not described in the mouse transcriptome analysis [13]. Conversely, SARS2 is down-regulated by JEV and HARS2 is down-regulated by WNV. Finally, two proteins not matching with the microarray hit study but involved in glutamate metabolism were modulated in our study: GLUL and ASNS, which are both up-regulated while glutamate metabolism was described as down-regulated in the microarray study.

Interestingly, we observed in our study a strong down-regulation of proteins associated with the extracellular matrix organization, collagen and cell-cell adhesion in both JEV- and WNV-infected neuroblastoma cells. This observation is consistent with the destruction of the extracellular matrix and collagen IV by MMP9 metalloprotease previously described for both JEV in rat astrocytes and WNV in mouse brain [43,44]. It is worth to note that the only metalloproteases identified in our study were all down-regulated: MIPEP, YME1L, ECE1 and MME.

PAM and SSBP2, the most down-regulated proteins by JEV and WNV infections, were not assigned to any functional cluster or connected to the network of proteins modulated during either JEV or WNV infection. To our knowledge, this is the first report of a link between JEV or WNV and expression of these two proteins. PAM is a key enzyme for the activation of neuropeptides [45], which immuno-modulatory effects are important for infection by other neurotropic viruses [46,47]. Thus, by targeting PAM expression, JEV and WNV could modulate the host response to viral infection. Furthermore, down-regulation of SSBP2 expression, a protein notably involved in RNA transcription, could be linked to the control of gene expression during flavivirus infection [48]. This is also consistent with modulation of the expression of several genes involved in transcription regulation and mRNA processing by JEV (RPRD2, POLR2A and CD2BP2), by JEV and WNV (YBX1, BCAS2, FRG1) or only by WNV (SYNCRIP). Interestingly, phosphorylation of YBX1 and POLR2A was modulated during WNV infection in a separate study [12].

The role of mosquito saliva in arbovirus infection is well-established [18,49,50], and while it is well-established for WNV [24], it has yet to be confirmed for JEV. Under the hypothesis that various factors from mosquito saliva may be able to reach the BBB and CNS, either independently or bound to the virus [40], our data suggest that mosquito saliva has the capacity to strongly affect JEV and WNV neuropathogenesis. Mosquito saliva alone has a low impact on neuroblastoma cells, only decreasing the expression of a group of proteins involved in nucleocytoplasmic transport, potentially modulating the nuclear translocation of JEV and WNV proteins [51] or the global host response to viral infection [52]. Interestingly, the differences in protein expression in JEV and WNV infected cells which are distinct between SGE-treated and untreated cells suggest a synergy between the virus and the saliva to disturb cellular homeostasis. While mosquito saliva mostly modulates transcription regulation-related proteins in association with JEV infection, it essentially modulates protein maturation and trafficking in association with WNV infection. Although several studies reported an immune response inhibition by mosquito saliva to WNV infection and especially of TNFα [53] or IFN [54], our study does not establish a specific down-regulation of their signaling in our human neuron model.

## Conclusion

To the best of our knowledge, we provide here the first proteomic comparison of JEV or WNV infected human neuroblastoma cells using a label-free quantification of whole-cell extract approach. Major host functions such as immune response, extracellular matrix organization, collagen metabolism, transcription regulation and mRNA processing are highly modulated during both JEV and WNV infections. Furthermore, mosquito saliva appears to have a strong impact on the infection of neuroblastoma cells by JEV or WNV. To confirm the capacity and depth of modulation of neurotropic flavivirus infection of the CNS by mosquito saliva, new studies should focus on advanced cell culture models combining a BBB to a compartment reflecting the CNS complexity *in vitro*. Furthermore, it would be interesting to compare the effects of neurotropic flaviviruses or non-flavivirus neurotropic viruses on the proteome of infected cells in order to determine their specificity.

## Supporting information

S1 Fig Hierarchical clustering of the LC-MS/MS data. The dendrogram was obtained in Perseus v1611.
(PDF)

S2 Fig Control of SK-N-SH cell infection by JEV or WNV. Viral titer (A) and protein quantification (B) of the infection in the samples processed by mass spectrometry.
(PDF)

S3 Fig Network analysis of proteins modulated during JEV infection in presence of SGE compared to untreated mock cells. Networks of up(red)- and down(green)-regulated proteins. PPI networks were determined with STRING and visualized with Cytoscape. Proteins regulated in common with JEV are highlighted in bold. Node size is relative to the number of edges. Edges are determined according to the number of sources (text mining, experiments, databases or co-expression) supporting the link between proteins.
(PDF)

S4 Fig Network analysis of proteins modulated during WNV infection in presence of SGE compared to untreated mock cells. Networks of up(red)- and down(green)-regulated proteins. PPI networks were determined with STRING and visualized with Cytoscape. Proteins regulated in common with WNV are highlighted in bold. Node size is relative to the number of edges. Edges are determined according to the number of sources (text mining, experiments, databases or co-expression) supporting the link between proteins.
(PDF)

S1 Table Complete list of proteins identified by label-free MS and up-regulated during JEV or WNV infection. Proteins are sorted according to the fold change in the JEV- (or secondly in WNV-) infected cells compared to the Mock.
(XLSX)

S2 Table Complete list of proteins by label-free MS and down-regulated during JEV or WNV infection. Proteins are sorted according to the fold change in the JEV- (or secondly in WNV-) infected cells.
(XLSX)

S3 Table Functional clusters of the modulated proteins during JEV infection. (XLSX)

S4 Table Functional cluster of the modulated proteins during WNV infection. (XLSX)

S5 Table Complete list of proteins identified by label-free MS and up-regulated in the presence of mosquito SGE during JEV or WNV infection. Proteins are sorted according to the fold change in the JEV- (or secondly in WNV-) infected cells compared to untreated cells.
(XLSX)

S6 Table Complete list of proteins identified by label-free MS and down-regulated in the presence of mosquito SGE during JEV and WNV infection. Proteins are sorted according to the fold change in JEV- (or secondly in WNV-) infected cells compared to untreated cells.
(XLSX)

S7 Table Functional clusters of the proteins modulated by the presence of mosquito SGE in JEV infected cells. (XLSX)

S8 Table Functional clusters of the proteins modulated by the presence of mosquito SGE in WNV infected cells. (XLSX)

S9 Table Complete list of proteins identified by label-free MS and up-regulated during JEV or WNV infection in the presence of mosquito SGE compared to mock in the absence of SGE. Proteins are sorted according to the fold change of the JEV- (or secondly to WNV-) infected cells compared to the untreated mock.
(XLSX)

S10 Table Complete list of proteins identified by label-free MS and down-regulated during JEV or WNV infection in the presence of mosquito SGE compared to mock in the absence of SGE. Proteins are sorted according to the fold change of the JEV- (or secondly to WNV-) infected cells compared to the untreated mock.
(XLSX)

S1 Raw images (PDF)
