# Supplementary material for: Comparison of a human neuronal model proteome upon Japanese encephalitis or West Nile Virus infection and potential role of mosquito saliva in neuropathogenesis
Source: PLoS One. 2020 May 6;15(5):e0232585. doi: 10.1371/journal.pone.0232585 (PMC7202638; doi:10.1371/journal.pone.0232585)
Supplement: S1 Fig — The dendrogram was obtained in Perseus v1611. (PDF) [file pone.0232585.s001.pdf]

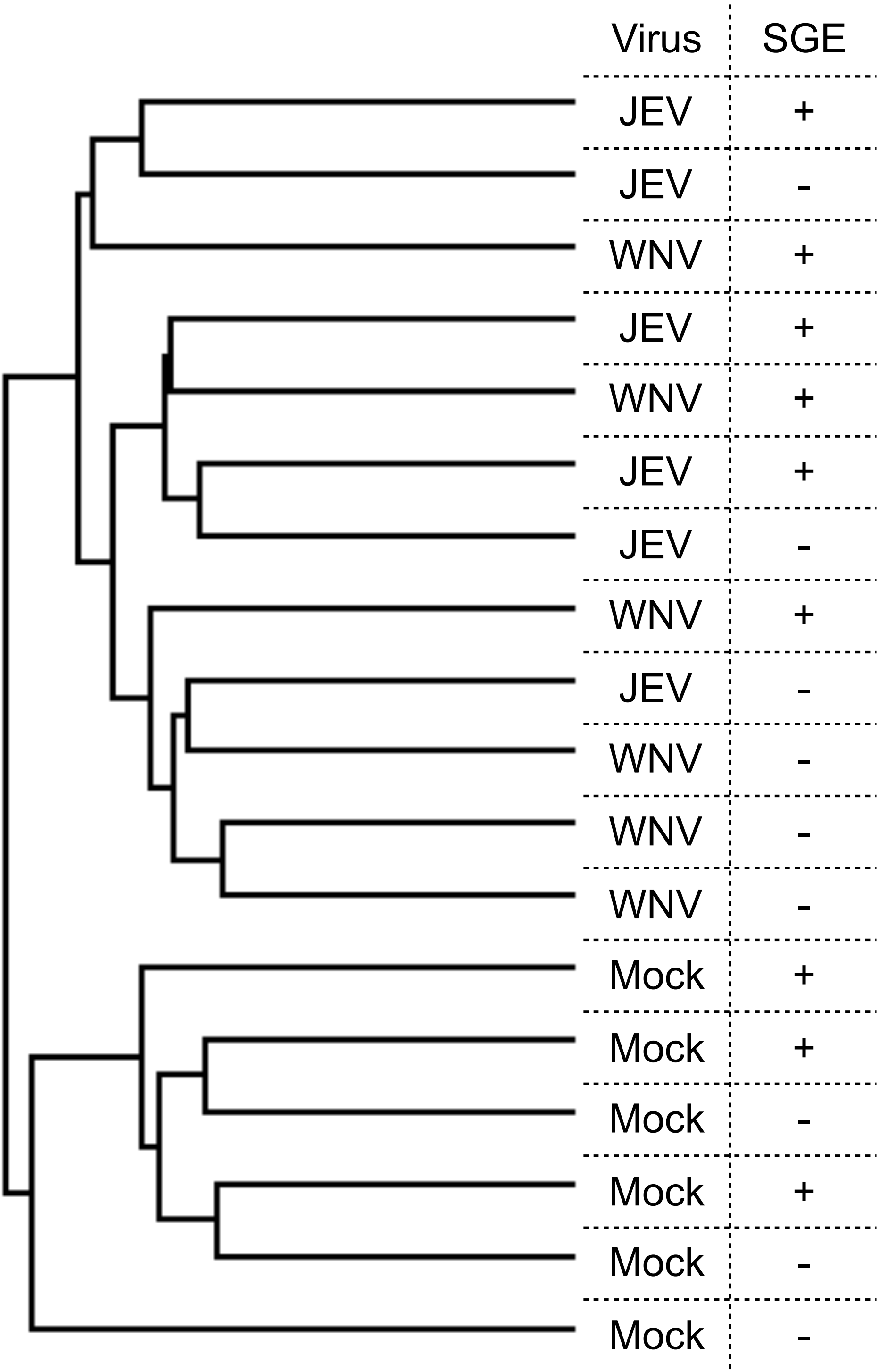

Virus
SGE
JEV
+
JEV
-
WNV
+
JEV
+
WNV
+
JEV
+
JEV
-
WNV
+
JEV
-
WNV
-
WNV
-
WNV
-
Mock
+
Mock
+
Mock
-
Mock
+
Mock
-
Mock
-
