# Supplementary figures and images for: Comparison of a human neuronal model proteome upon Japanese encephalitis or West Nile Virus infection and potential role of mosquito saliva in neuropathogenesis
Source: PLoS One. 2020 May 6;15(5):e0232585. doi: 10.1371/journal.pone.0232585 (PMC7202638; doi:10.1371/journal.pone.0232585)

**A**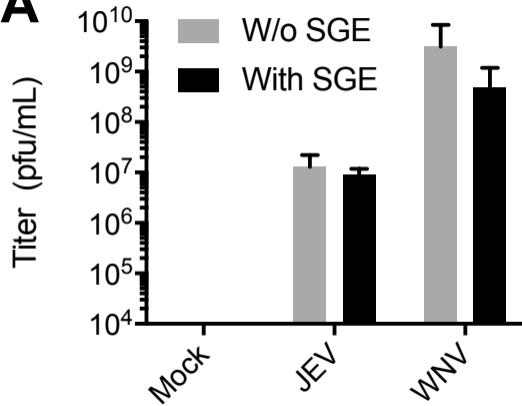**B**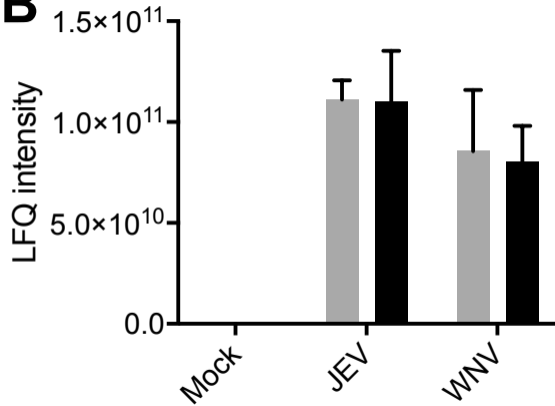

Supplement: S2 Fig — Viral titer (A) and protein quantification (B) of the infection in the samples processed by mass spectrometry. (PDF) [file pone.0232585.s002.pdf]

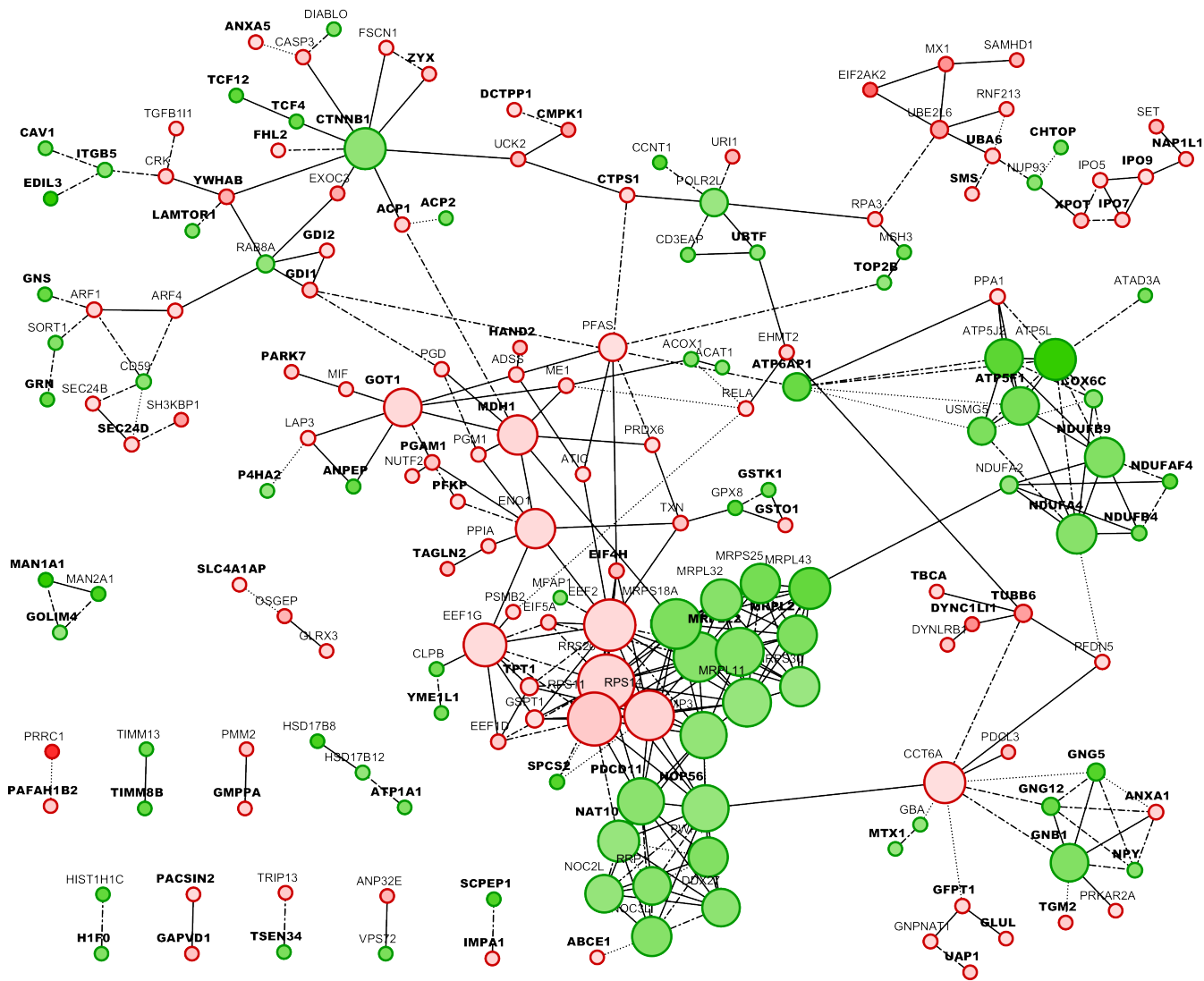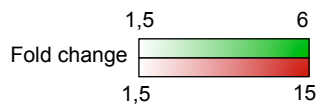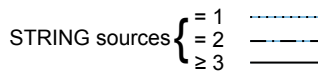

Supplement: S3 Fig — Networks of up(red)- and down(green)-regulated proteins. PPI networks were determined with STRING and visualized with Cytoscape. Proteins regulated in common with JEV are highlighted in bold. Node size is relative to the number of edges. Edges are determined according to the number of sources (text mining, experiments, databases or co-expression) supporting the link between proteins. (PDF) [file pone.0232585.s003.pdf]

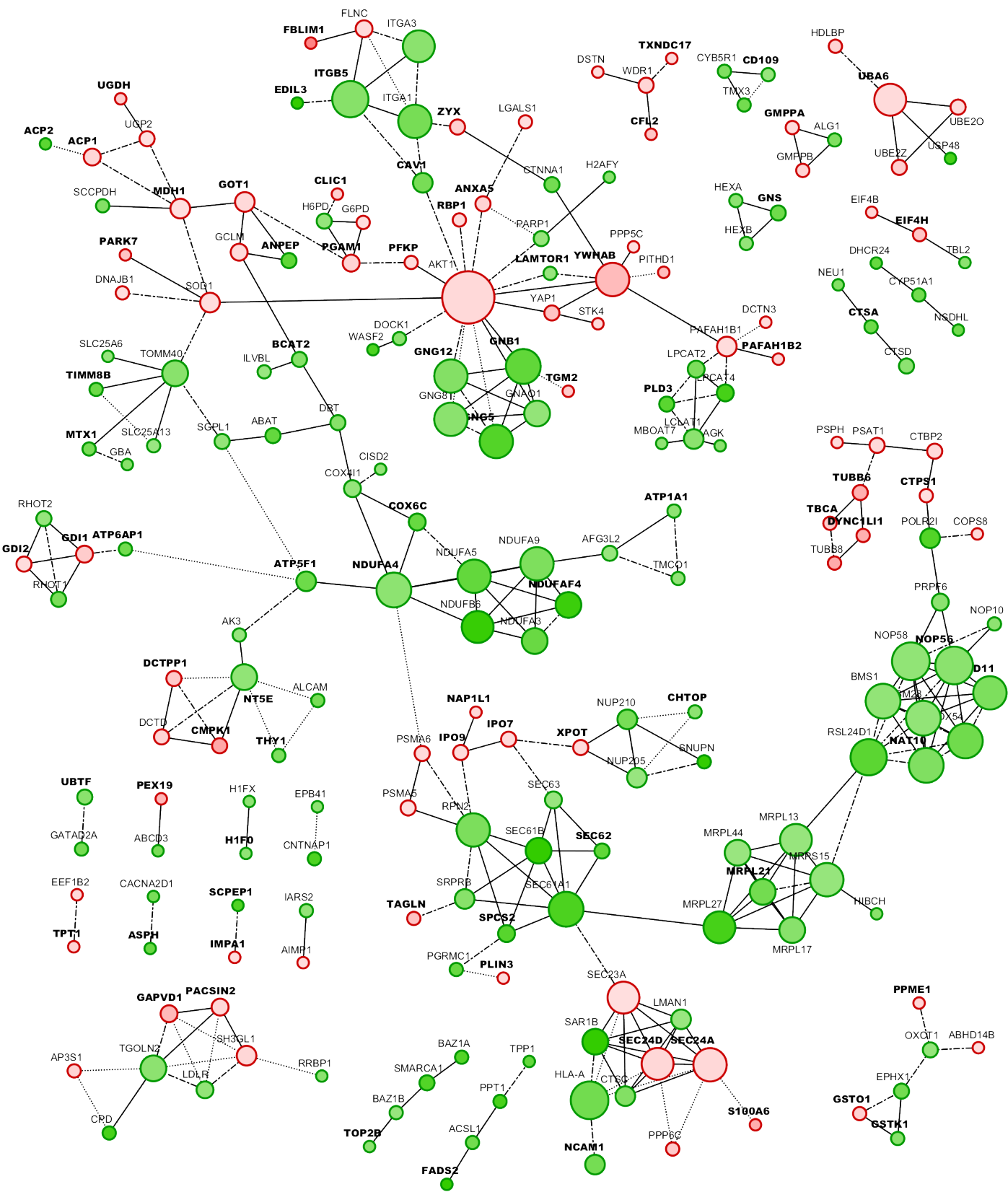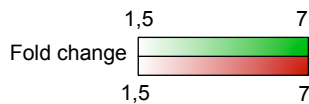

STRING sources {

- = 1
- = 2
- ≥ 3

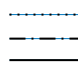

Supplement: S4 Fig — Networks of up(red)- and down(green)-regulated proteins. PPI networks were determined with STRING and visualized with Cytoscape. Proteins regulated in common with WNV are highlighted in bold. Node size is relative to the number of edges. Edges are determined according to the number of sources (text mining, experiments, databases or co-expression) supporting the link between proteins. (PDF) [file pone.0232585.s004.pdf]
