# Supplementary material for: Comparison of a human neuronal model proteome upon Japanese encephalitis or West Nile Virus infection and potential role of mosquito saliva in neuropathogenesis
Source: PLoS One. 2020 May 6;15(5):e0232585. doi: 10.1371/journal.pone.0232585 (PMC7202638; doi:10.1371/journal.pone.0232585)

E protein

Exposure: 5m49s

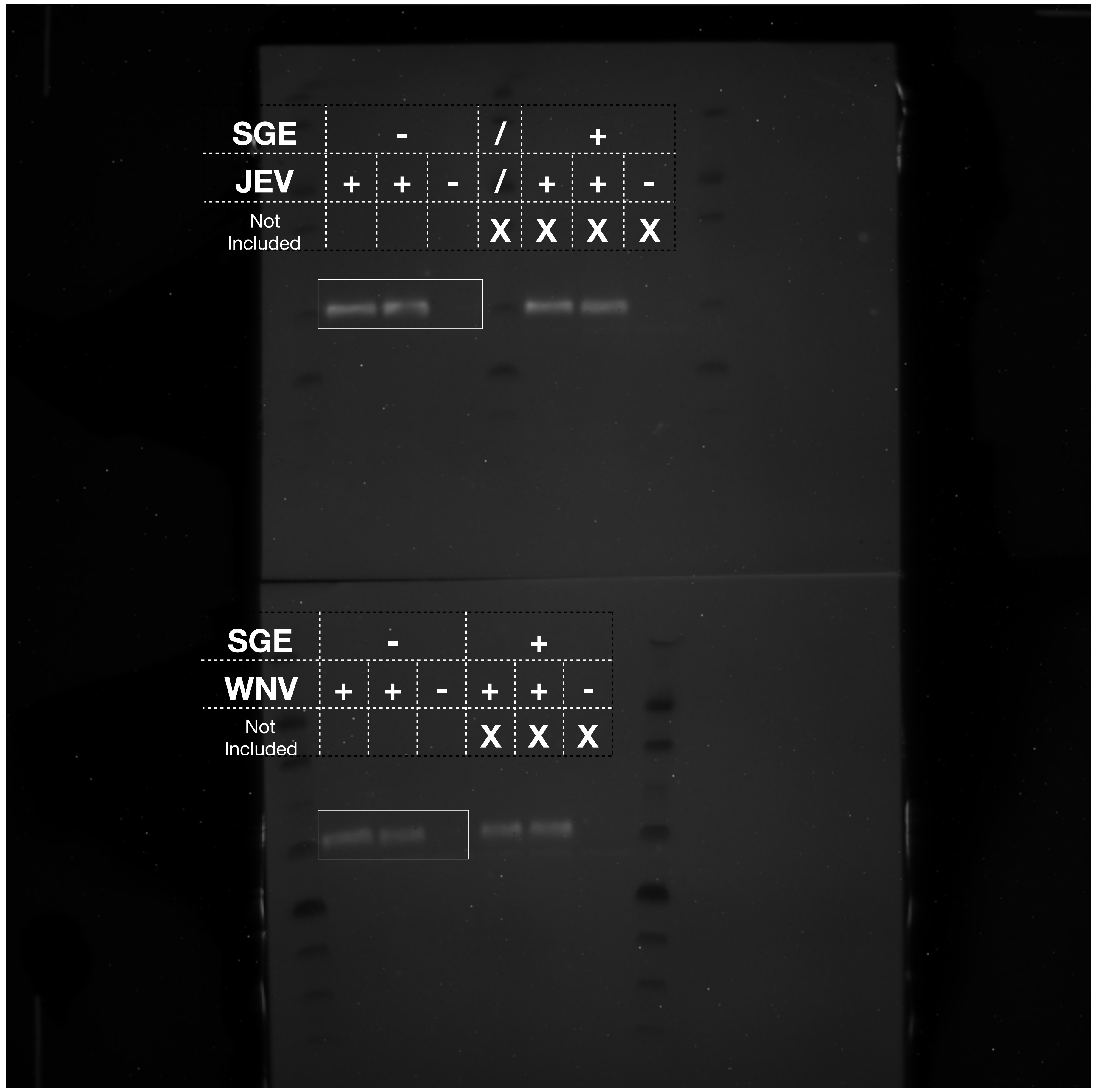

Actin

Exposure: 1m27s

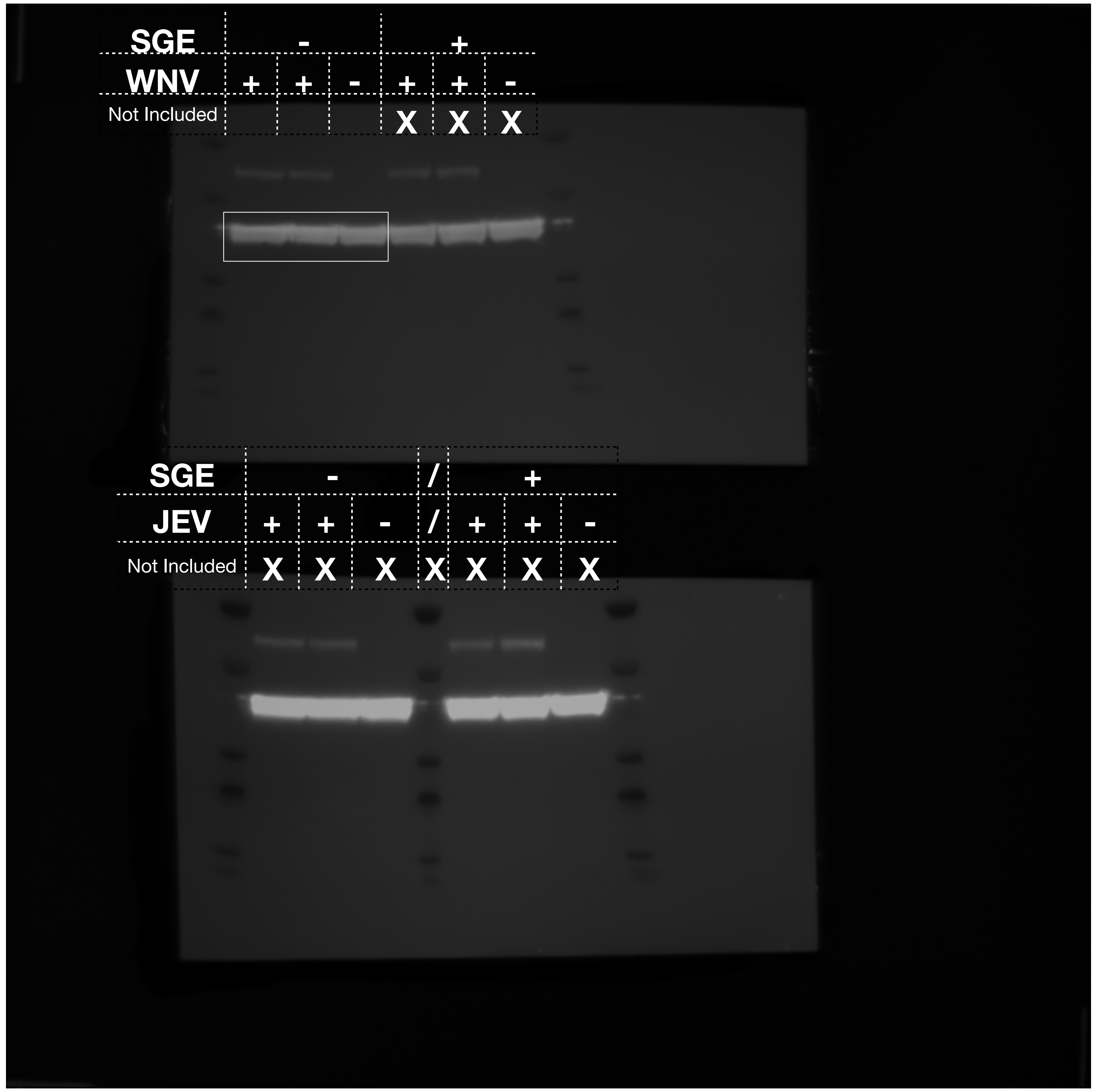

Exposure: 30s

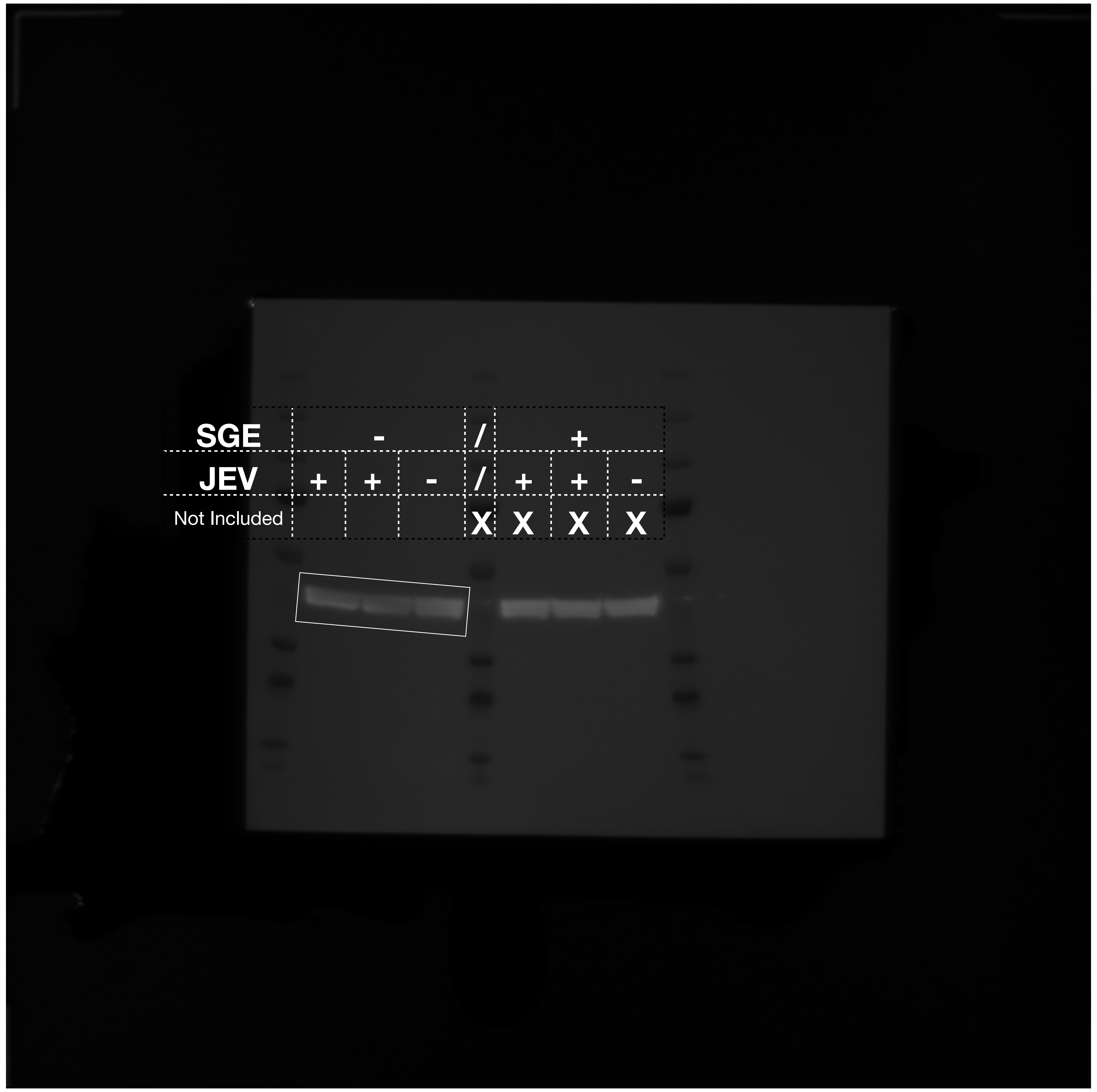

COL1A1

Exposure: 30m48s

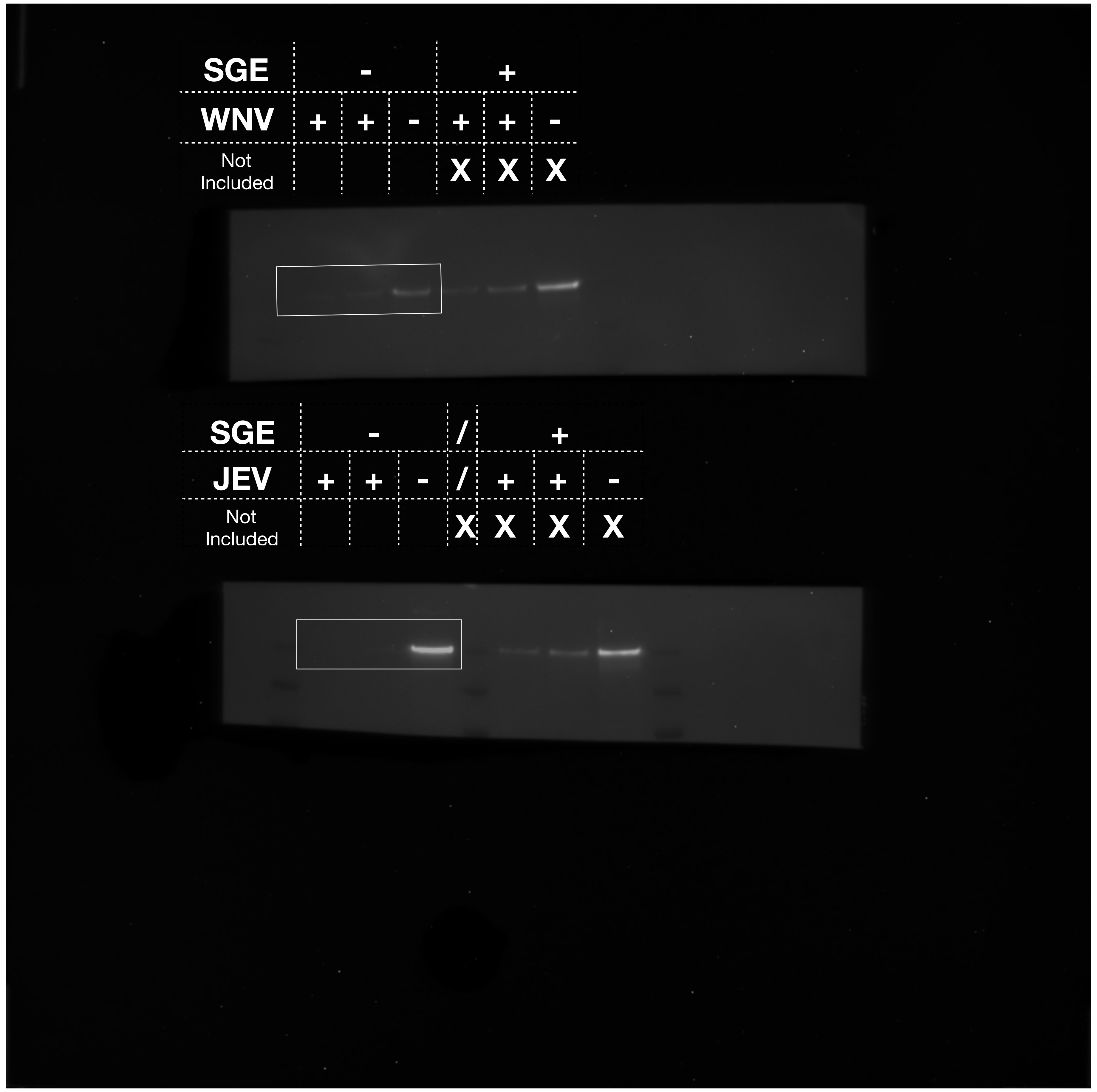

IFIT3

Exposure: 4m56s

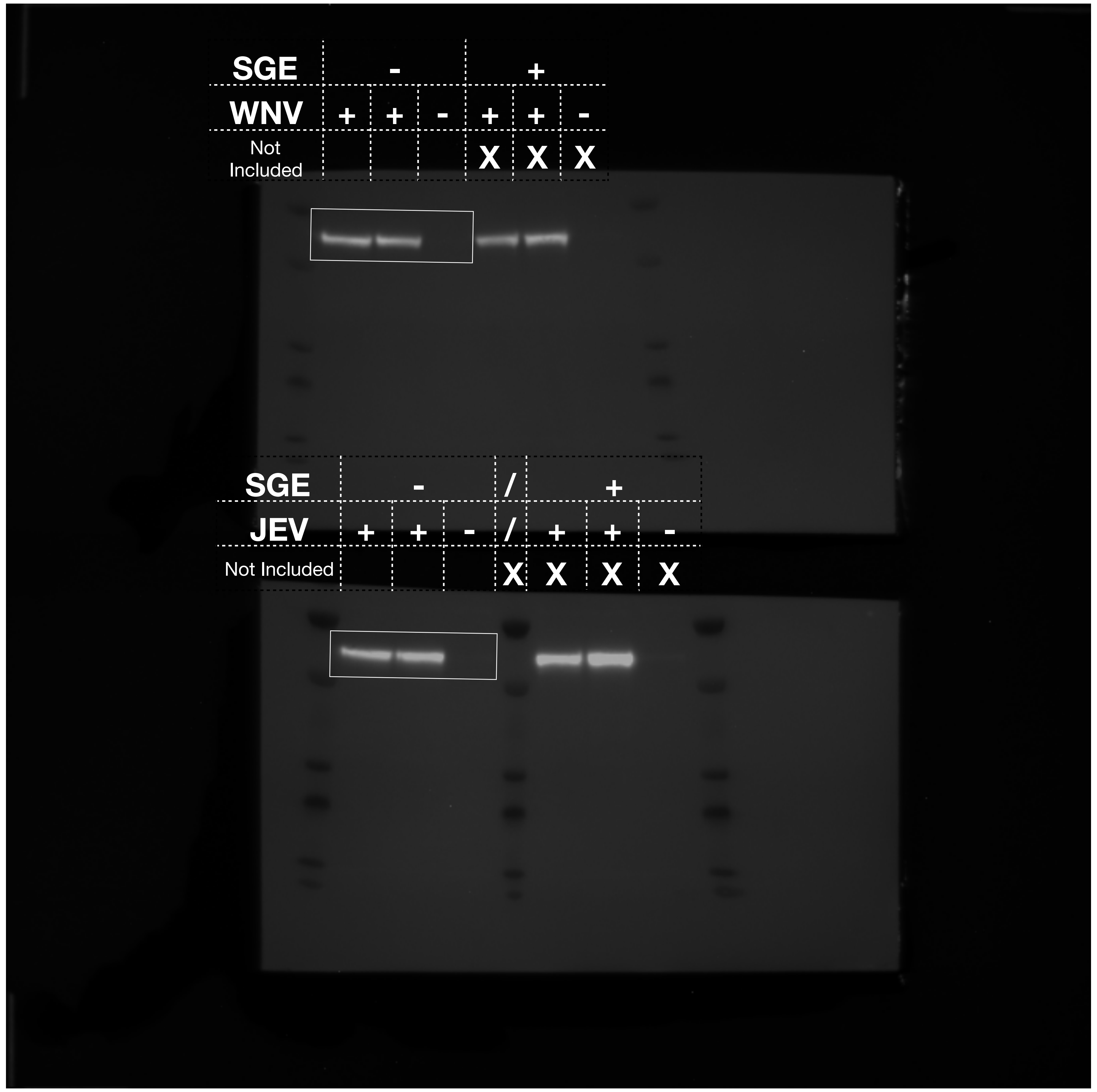

PAM

Exposure: 1h30m33s

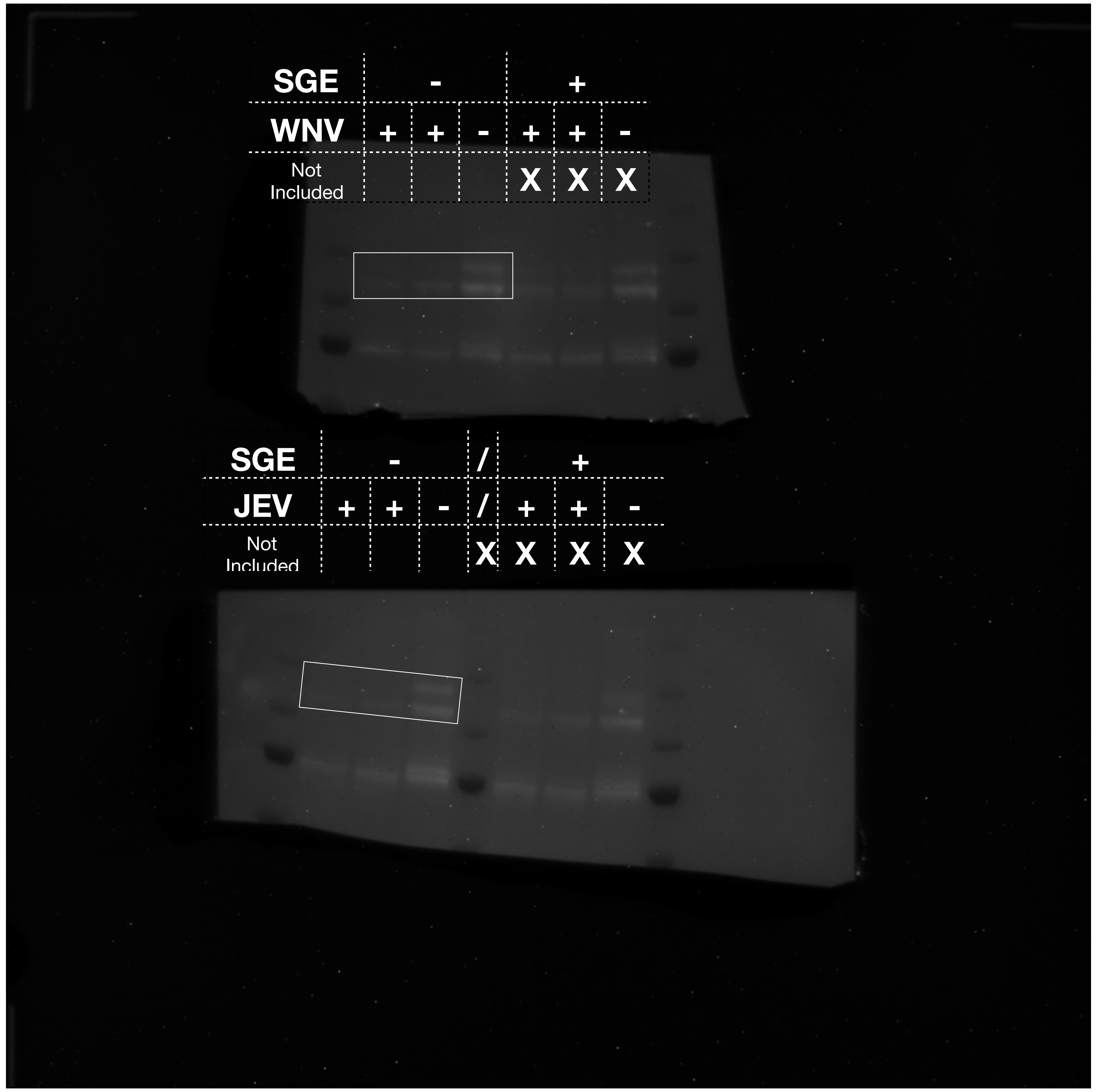

SSBP2

Exposure: 1h15m19s

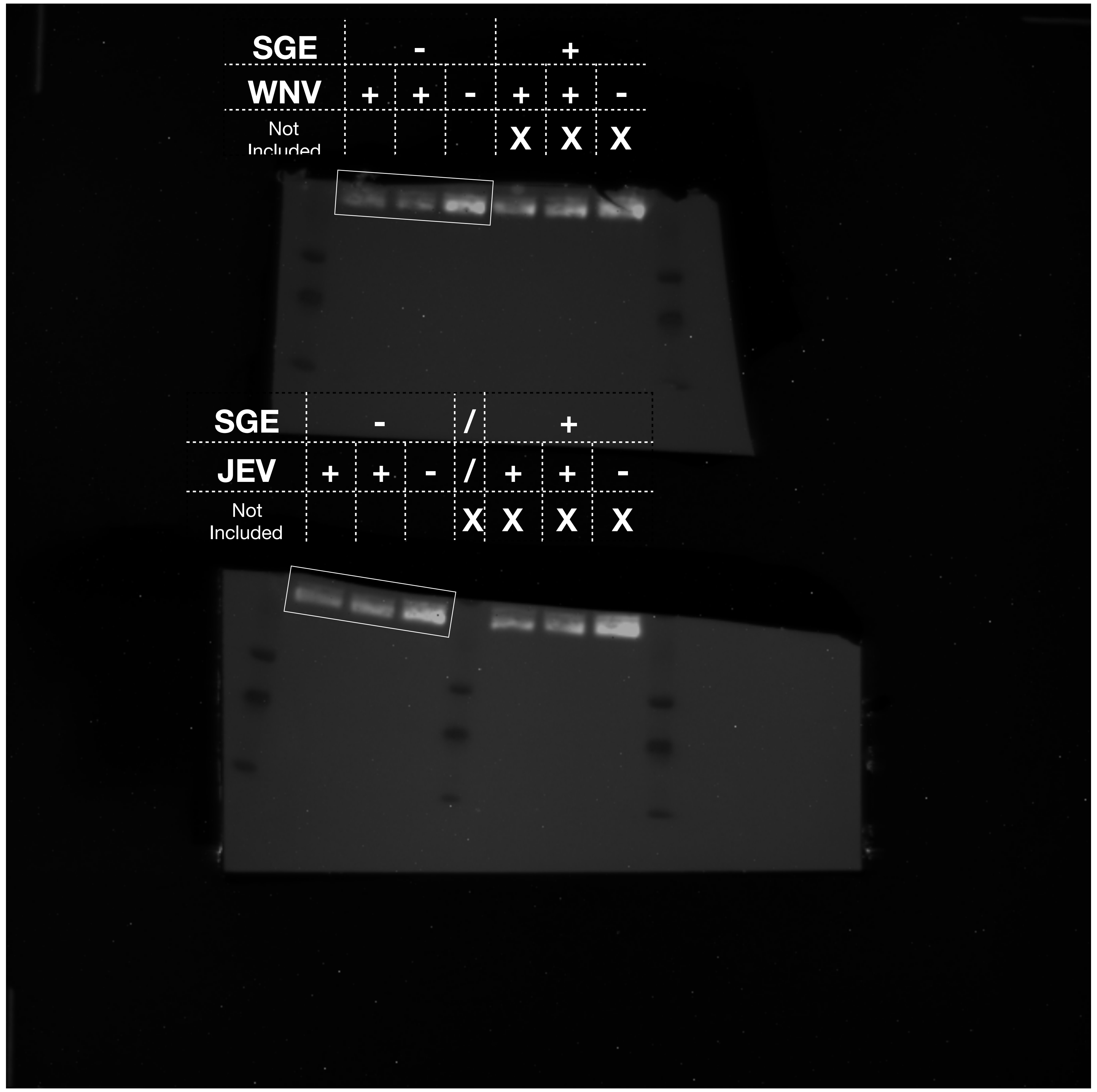

Supplement: S1 Raw images — (PDF) [file pone.0232585.s015.pdf]
